# miR-182 Regulates Slit2-Mediated Axon Guidance by Modulating the Local Translation of a Specific mRNA

**DOI:** 10.1016/j.celrep.2016.12.093

**Published:** 2017-01-31

**Authors:** Anaïs Bellon, Archana Iyer, Simone Bridi, Flora C.Y. Lee, Cesaré Ovando-Vázquez, Eloina Corradi, Sara Longhi, Michela Roccuzzo, Stephanie Strohbuecker, Sindhu Naik, Peter Sarkies, Eric Miska, Cei Abreu-Goodger, Christine E. Holt, Marie-Laure Baudet

**Affiliations:** 1 PDN Department, University of Cambridge, Cambridge CB23DY, UK; 2 CIBIO, University of Trento, Trento 38123, Italy; 3 Unidad de Genómica Avanzada (Langebio), Cinvestav, Irapuato 36821, Mexico; 4 Gurdon Institute, University of Cambridge, Cambridge CB21QN, UK

**Keywords:** miRNAs, local translation, growth cone, axon guidance, brain wiring, miR-182, Slit2, cofilin

## Abstract

During brain wiring, cue-induced axon behaviors such as directional steering and branching are aided by localized mRNA translation. Different guidance cues elicit translation of subsets of mRNAs that differentially regulate the cytoskeleton, yet little is understood about how specific mRNAs are selected for translation. MicroRNAs (miRNAs) are critical translational regulators that act through a sequence-specific mechanism. Here, we investigate the local role of miRNAs in mRNA-specific translation during pathfinding of *Xenopus laevis* retinal ganglion cell (RGC) axons. Among a rich repertoire of axonal miRNAs, miR-182 is identified as the most abundant. Loss of miR-182 causes RGC axon targeting defects in vivo and impairs Slit2-induced growth cone (GC) repulsion. We find that miR-182 targets cofilin-1 mRNA, silencing its translation, and Slit2 rapidly relieves the repression without causing miR-182 degradation. Our data support a model whereby miR-182 reversibly gates the selection of transcripts for fast translation depending on the extrinsic cue.

## Highlights

• Small RNA-seq analysis reveals that miR-182 is the most abundant miRNA in RGC axons
• miR-182 regulates Slit2-mediated axon guidance of RGCs in vitro and in vivo
• miR-182 silences cofilin-1 local protein synthesis in growth cones
• Slit2 rapidly lifts miR-182-mediated repression of cofilin-1 without degrading it

## Graphical Abstract

**Figure:**
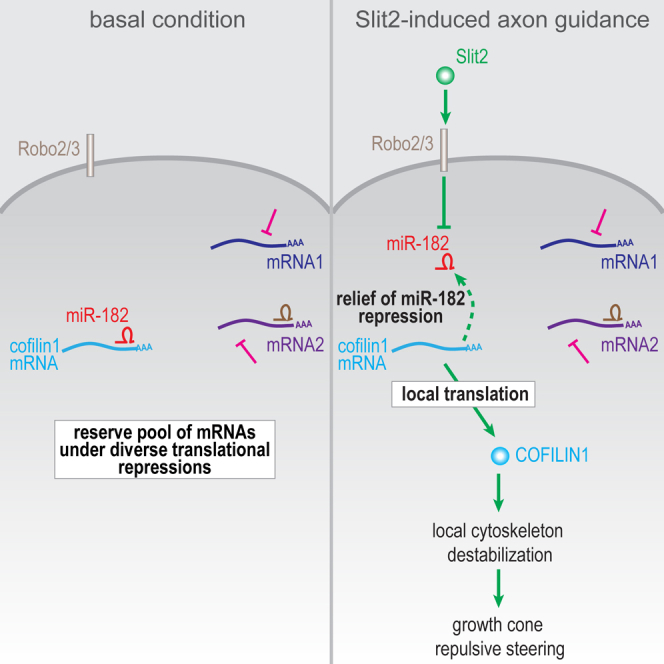

## Teaser

Bellon et al. examine how specific mRNAs are selected for cue-induced local protein synthesis during axon guidance and find that miR-182 reversibly regulates the selective translation of a specific transcript to facilitate fast growth cone steering and axon guidance.

## Introduction

The accurate wiring of the nervous system depends on the ability of axons to extend from neuronal somata to reach their specific synaptic targets during development. Growth cones (GCs) lead growing axons to their correct destinations by responding directionally to attractive and repulsive cues encountered along the pathway (Bouquet and Nothias, 2007). Given the extreme distance that can separate pre- and post-synaptic populations of neurons, axon pathfinding presents a unique challenge for neurons in ensuring that GCs respond properly and rapidly to guidance stimuli. During recent years, it has become clear that axons and GCs possess a high degree of functional autonomy and that this is aided by local protein synthesis (LPS) (Holt and Schuman, 2013). A complex and changing repertoire of mRNAs is trafficked into growing axons and GCs (Zivraj et al., 2010, Gumy et al., 2011, Gumy et al., 2014), where some are locally translated in response to guidance cues independent of cell bodies (Campbell and Holt, 2001, Brittis et al., 2002, Yao et al., 2006, Lin and Holt, 2007). Studies investigating LPS regulation in axons have linked guidance signaling with the regulation of global translational activity in the GC, such as the activation of the initiation factor eIF-4E (eukaryotic initiation factor 4E) (Campbell and Holt, 2001, Piper et al., 2006), or the sequestration of ribosomal components (Tcherkezian et al., 2010). However, evidence points to a selective model of translation whereby specific subsets of mRNAs from a complex mRNA pool (Lin and Holt, 2007, Deglincerti and Jaffrey, 2012) are differentially translated in response to different extrinsic cues while others remain translationally silent. For example, Slit2 and Semaphorin3A (Sema3A) mediate GC repulsion via the translation of cofilin-1 (Cfl1) and RhoA (Piper et al., 2006, Wu et al., 2005), respectively, whereas Netrin-1 and brain-derived neurotrophic factor (BDNF) promote attraction by the local synthesis of β-actin (Leung et al., 2006, Yao et al., 2006). A major unresolved question is how a given transcript is specifically selected for translation in GCs in response to a given guidance cue.

Although extrinsic cues facilitate mRNA-specific translation in GCs through the regulation of RNA-binding protein (RBP)-mediated axonal transport (Vuppalanchi et al., 2009), no mechanisms directly regulating the translation of specific mRNAs in the GC have been identified so far for directional steering. Moreover, given the complex nature of mRNA translation in developing axons (Shigeoka et al., 2013), RBPs alone are unlikely to account fully for the complex regulation of mRNA-specific translation in GCs during guidance, and additional layers of regulation are probably involved.

MicroRNAs (miRNAs) have emerged as key translational regulators possessing mRNA target specificity. miRNAs are first transcribed as long primary molecules, pri-miRNAs, and then processed by Drosha and Dicer to generate mature miRNA molecules (Kim et al., 2009). These non-coding ∼21 nt long molecules bind to complementary sequences on mRNAs (Bartel, 2009) and modulate their stability and/or translation (Bazzini et al., 2012, Djuranovic et al., 2012, Eichhorn et al., 2014). Due to the sequence-specific regulation of mRNA translation by miRNAs, one way to control mRNA-specific translation during axon guidance could be regulation by miRNAs. Several lines of evidence suggest that miRNAs are involved in axon guidance and GC steering, but their mechanism of action remains poorly understood (Iyer et al., 2014). First, in mouse, the absence of Dicer induces severe axon pathfinding defects in the visual pathway in vivo (Pinter and Hindges, 2010). Second, in *Xenopus* retinal axons, miR-124 regulates the onset of expression of neuropilin1 (Sema3A receptor) and controls a Sema3A-mediated guidance decision in vivo (Baudet et al., 2011). Finally, miR-134 is required in *Xenopus* spinal neurons for BDNF-induced GC steering in vitro (Han et al., 2011). miRNAs (Hancock et al., 2014, Natera-Naranjo et al., 2010, Sasaki et al., 2014) and the functional RNA-induced silencing complex (RISC) (Hengst et al., 2006) have been shown to reside in developing axons, suggesting that miRNAs may act locally within this neuronal compartment.

Here we have investigated whether miR-182, identified from an axonal profiling screen, can regulate the guidance of *Xenopus* retinal ganglion cell (RGC) axons in the visual pathway by modulating the axonal translation of specific mRNAs. We show that miR-182 depletion causes RGC axon targeting defects in vivo that phenocopy Slit2 knockdown in the brain. In the absence of miR-182, protein synthesis-dependent GC repulsive steering in response to Slit2 is abolished. Furthermore, we demonstrate that miR-182 directly targets Cfl1 mRNA, a key cytoskeleton regulator, and is required for Slit2-induced axonal Cfl1 synthesis. Finally, we show that Slit2 inhibits the activity of miR-182 in GCs, without degrading it. We propose that under basal conditions, axonal miR-182 represses the de novo synthesis of Cfl1 in the GC. Upon Slit2 stimulation, miR-182 is inactivated, temporarily relieving Cfl1 mRNA from its repression and allowing its local translation, which facilitates the cytoskeletal changes that underlie directional steering.

## Results

### Growing RGC Axons Contain a Rich Repertoire of miRNAs

**Figure 1:**
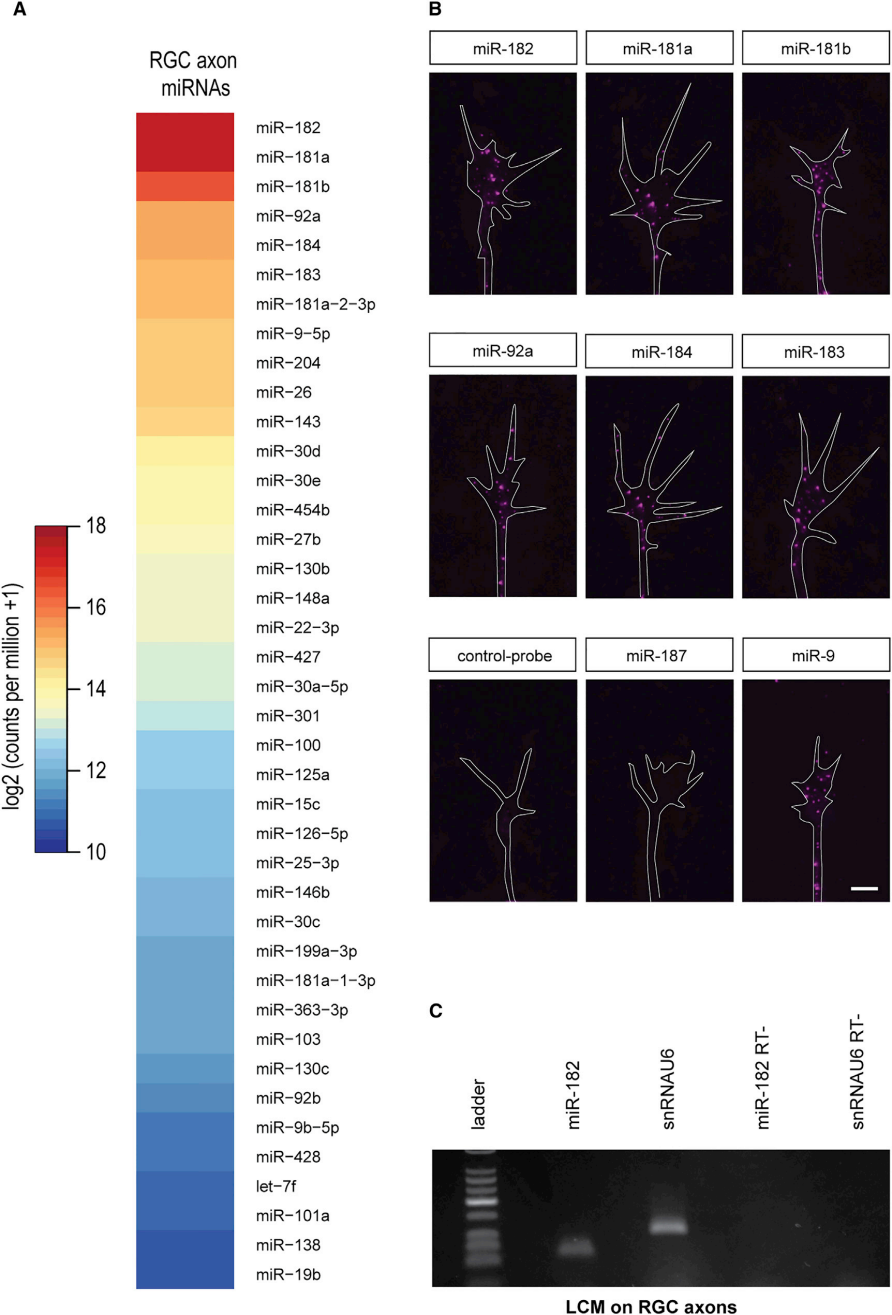
miR-182 Is Localized in RGC Axons (A) Heatmap representing the average expression of mature miRNAs from two axonal small RNA-sequencing (sRNA-seq) libraries prepared from stage 37/38 retinal cultures. The figure is sorted by decreasing axonal average values. (B) Fluorescent ISH on stage 35/36 RGC GCs cultured in vitro for 24 hr. (C) TaqMan qPCR performed on RNA extracted from laser-captured stage 37/38 RGC axons. U6 snRNA was used as positive control, because it is found in developing axons (Natera-Naranjo et al., 2010, Zhang et al., 2013, Hancock et al., 2014). RT−, no template negative control; snRNAU6, U6 snRNA. Scale bar, 5 μm (B). See also Figure S1 and Table S1.

To characterize the full repertoire of miRNAs in developing RGC axons, we performed an unbiased analysis of miRNAs residing in the axonal compartment using Illumina Next-Generation Sequencing technology. To obtain sufficient axonal material, 1,000 eyes from stage 37/38 (according to Nieuwkoop and Faber, 1994) *Xenopus* larvae were cultured for 48 hr for each experiment. Intact eyes were explanted with the optic nerve exit point (back of eye) positioned in contact with the culture substrate to facilitate the outgrowth, exclusively, of RGC axons. RGC axons were subsequently harvested from the culture substrate by manual removal of the explanted eyes (Figures S1A and S1B). This approach has been used previously to successfully obtain pure axon material (Yoon et al., 2012). The purity of the axonal material was validated by RT-PCR, which showed the presence of β-actin mRNA, known to be expressed in developing axons (Leung et al., 2006), and the absence of microtubule-associated protein 2 (MAP2) transcript, whose expression is known to be restricted to cell bodies and dendrites (Figure S1C) (Kleiman et al., 1990). Libraries from two biological replicates of 22–30 nt gel-excised small RNAs were sequenced. The two libraries yielded 7.8 and 10.8 million reads and revealed the presence of 148 miRNAs in growing RGC axons, with at least 1 read in both replicates (Figure 1A; Table S1). The two replicates were highly correlated, as judged by the expression level of all miRNAs (Pearson’s correlation coefficient = 0.93) (Figure S1D). The most abundant miRNAs detected were miR-182, miR-181a, miR-181b, miR-92a, miR-184, and miR-183, representing 25%, 17.8%, 7.9%, 4.6%, 3.9%, and 3.8%, respectively, of the total miRNAs in developing RGC axons (Figure 1A). In situ hybridization (ISH) experiments were performed to validate the sequencing results. We successfully detected the presence of an ISH signal in cultured RGC axons and GCs for the 15 most abundant sequenced axonal miRNAs, as well as for the brain-specific miRNA miR-9 (Figure 1B) (data not shown). In contrast, no signal was detected when using a control probe or a probe against miR-187, a miRNA not detected in RGC axons by sequencing (Figure 1B).

Analysis of the RNA sequencing (RNA-seq) results identified miR-182 as the most abundant axonal miRNA. Its presence in axons was validated using ISH (as described earlier) (Figure 1B) and qPCR from axons collected by laser capture microdissection (LCM) (Figure 1C). Although miR-182 presence was undetectable in RGC soma through ISH in vivo (Figure S2B), the presence of miR-182 in axons suggests that it is, at least transiently, expressed in the RGC cell body. TaqMan qPCR, a more sensitive detection method, detected miR-182 in RGC soma in vivo, collected by LCM (average Ct: 27.65 ± 1.52; positive control U6 small nuclear RNA [snRNA], average Ct: 23.26 ± 0.61) (Figure S1E). In comparison with whole eye, miR-182 showed an average 8.0 ± 2.31-fold depletion in RGC soma using the ΔΔCt method, with U6 snRNA as a normalizer. Because eye cells also comprise many non-miR-182-expressing or poorly miR-182-expressing cells, this is a likely underestimation of the extent of miR-182 depletion in RGC soma compared to miR-182-expressing photoreceptor cells.

**Figure 2:**
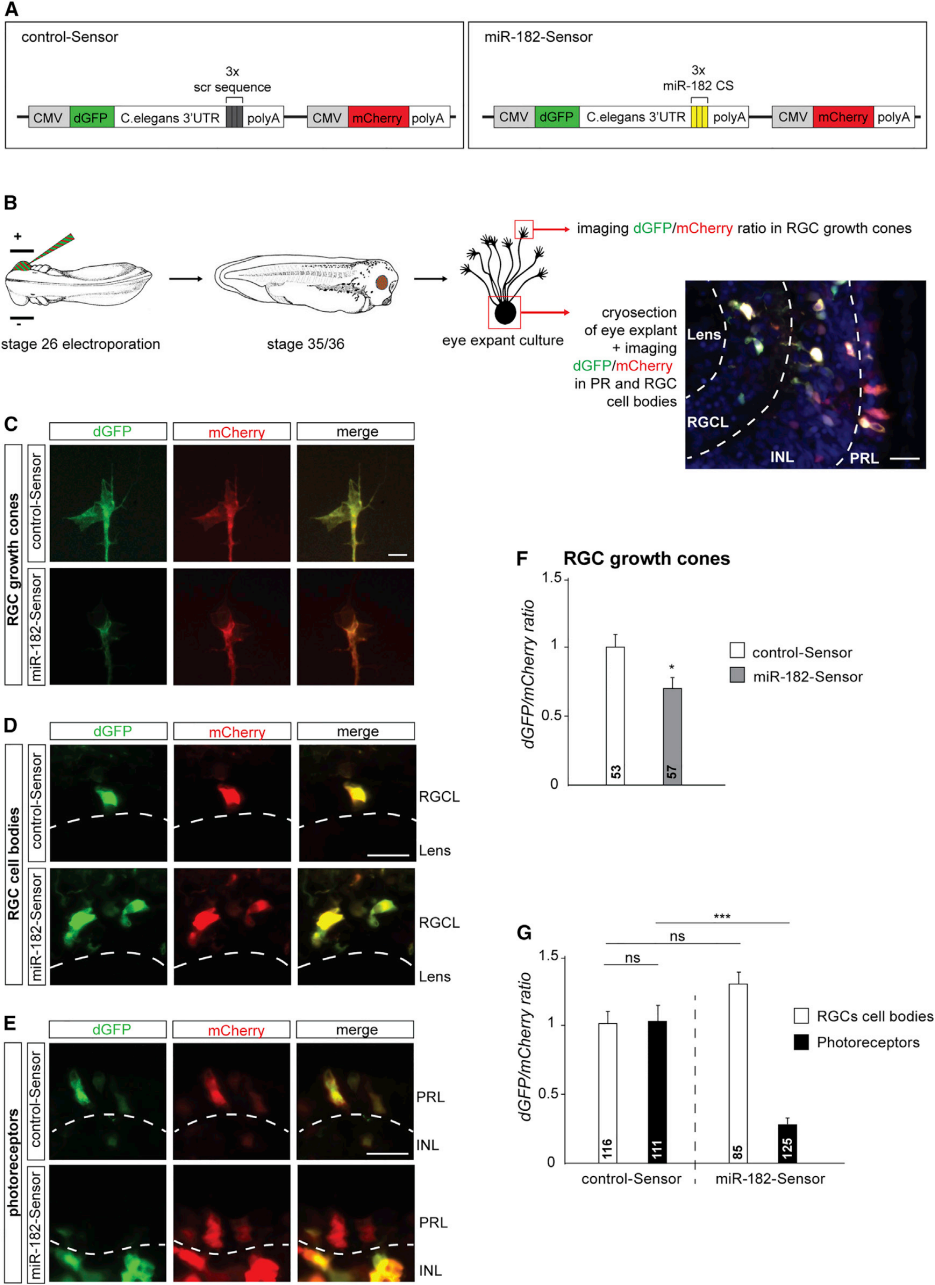
miR-182 Is Active and Enriched in RGC Axons (A) Sensor construct design. (B) Schematic representation of the experimental protocol. (C–E) Illustrative images of RGC GCs (C), RGC soma (D), or PRs (E) following retinal electroporation of control-Sensor or miR-182-Sensor. Clear examples of dGFP/mCherry ratio decrease are shown in (C) and (E). (F and G) Quantification of the dGFP/mCherry ratio at the RGC GCs, soma, or PRs. Values are mean ± SEM. Mann-Whitney test (F) and two-way ANOVA followed by Tukey post hoc test (G), ^∗^p < 0.05, ^∗∗∗∗^p < 0.0001. ns, nonsignificant; CMV, cytomegalovirus promoter; CS, complementary sequence; dGFP, destabilized GFP; INL, inner nuclear layer; PRL, photoreceptor layer; RGCL, retinal ganglion cell layer. Scale bars, 20 μm (B, D, and E) and 5 μm (C). See also Figure S2.

We next addressed whether miR-182 activity reflects its compartmentalized distribution using a reporter sensor of miRNA activity, similar in design to a previously used construct (De Pietri Tonelli et al., 2006). miR-182-Sensor expresses destabilized GFP (dGFP) under the regulation of a 3′ untranslated region (3′ UTR) containing three sequences complementary to miR-182, with mCherry as an internal control (Figure 2A). Any increase in miR-182 activity should lead to the decrease of dGFP while leaving mCherry expression levels unaltered. In control-Sensor, the three sequences complementary to miR-182 are replaced by scrambled sequences. It should thus be inert to change in miR-182 activity.

Sensor sensitivity was first validated in vivo in photoreceptors (PRs), where miR-182 is abundantly expressed (Figure S2B). Electroporation of sensors into stage 26 eyes and comparison of the dGFP/mCherry ratio in stage 41 retinas shows that the dGFP/mCherry ratio from miR-182-Sensor, but not from control-Sensor, is significantly decreased in PRs (−61% ± 0.02%) but not in amacrine-like cells (+1% ± 0.07%) (Figures S2A and S2C–S2E). This suggests that miR-182-Sensor specifically detects endogenous miR-182 activity in PRs in vivo but not in cells with no or low miR-182 expression.

To explore the compartmentalized action of miR-182 activity, we measured miR-182 activity in retinal explant-derived RGC soma and axons. The evaluation of local regulation of dGFP and mCherry transcripts in axons was possible, because dGFP and mCherry mRNAs are detected in this compartment (Figure S2F). Sensor-electroporated retinas were thus cultured at stage 35/36, and the fluorescence levels of dGFP and mCherry were measured directly in RGC GCs or in RGC soma and PRs of cryosectioned explants (Figure 2B). Quantification reveals that while the dGFP/mCherry ratio of control-Sensor remains unchanged between both cell types and compartments, the ratio of miR-182-Sensor is significantly decreased in RGC axons (−31% ± 8.1%) and PRs (−73.3% ± 0.04%) but not in RGC soma (+33.4% ± 0.11%) (Figures 2C–2G). This indicates that miR-182 is specifically active in the axonal compartment of RGCs but not in the soma.

Altogether, these results confirm the enrichment and activity of miR-182 in RGC axons and GCs and the reliability of our sequencing results.

### miR-182 Regulates Axon Targeting in the Optic Tectum In Vivo

To assess whether miR-182 plays a role in RGC axon guidance in vivo, we used a loss-of-function approach in the *Xenopus* visual system using miRNA antisense morpholino oligomers (MOs) and axon tracing. A miR-182 MO blocking the function of endogenous mature *Xenopus laevis* (xla) xla-miR-182 was injected into the dorsal blastomeres of eight-cell-stage embryos (Figure S3A). These two dorsal blastomeres are fated to give rise to the entire CNS; therefore, targeting them for MO delivery induces specific knockdown in the CNS, including the neural retina, at later stages (Leung and Holt, 2008). At stage 37/38, miR-182 morphants show almost no expression of miR-182 in the CNS by ISH. In contrast, control embryos show expression of miR-182 in the outer retina and different regions of the brain, such as the pineal gland, the otic vesicle, or the olfactory pit areas (Figure S3B), consistent with previously reported expression of miR-182 (Wei et al., 2015). This result indicates that injection of miR-182 MO at the eight-cell stage efficiently knocks down endogenous miR-182 until later developmental stages. No gross morphological defects were observed in miR-182 morphants (Figure S3A). The eye size and the number of RGCs, counted as Islet-1 positive/Sox2 negative cells on cryosections at stage 40 (Baudet et al., 2011), were similar to controls (Figures S3C–S3E). Altogether, these results indicate that the knockdown of miR-182 in the CNS does not affect the gross development of the eye or the maturation of RGCs.

**Figure 3:**
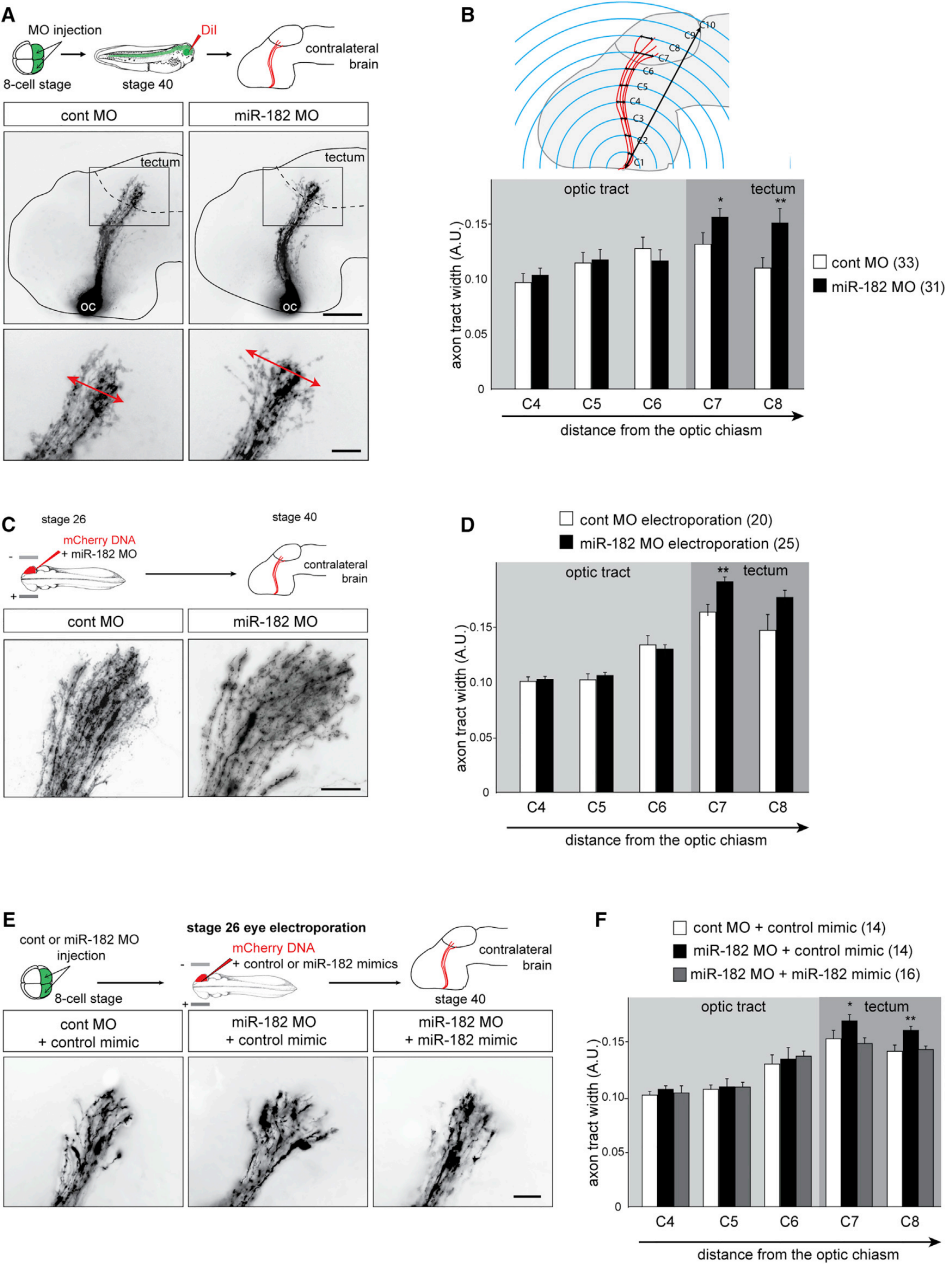
In Vivo, miR-182 Is Involved in RGC Axon Targeting but Not Long-Range Pathfinding (A, C, and E) Schematic representation of the experimental protocols and representative images of brains, where RGC axons are stained with DiI or expressing mCherry. Arrows delineate the width of the pathway (A). (B, D, and F) Quantification of pathway width. (B) Schematic representation of the methodology applied for pathway width measurements. Values are mean ± SEM. Numbers of brains analyzed are between brackets. Two-way ANOVA followed by Bonferroni post-test, ^∗^p < 0.05, ^∗∗^p < 0.01. Cont, control; MO, morpholino oligomer; RGC, retinal ganglion cell. Scale bars, 150 μm (A, top panels) and 50 μm (A, bottom panel; C; and E). See also Figure S3.

Next, we investigated whether miR-182 is involved in the pathfinding of RGC axons in vivo. During development, pioneering RGC axons exit the eye at stage 28, cross the optic chiasm at stage 32, and grow dorsally to project to their midbrain target, the optic tectum, at stage 37/38. By stage 40, most axons from the central retina have reached their final destination (Holt, 1989). miR-182 morphants and control embryos were raised to stage 40, and RGC axons were anterogradely labeled by lipophilic DiI filling of the eye (Figure 3A). In miR-182 morphant embryos, RGC axons project appropriately through the optic pathway on the contralateral side of the brain (Figure 3A), and no difference in RGC axon length is observed between control and miR-182 MO-injected embryos (Figures 3B, S3F, and S3G). This suggests that miR-182 is not essential for growth and long-range pathfinding of RGC axons to the tectal area. However, immediately after entering the tectum, the trajectories of the RGC axon terminals appear to disperse more widely in miR-182 morphants (Figure 3A, insets) with axons often straying aberrantly toward the dorsal midline. The width of the DiI-labeled RGC axon pathway was measured at regular intervals from the optic chiasm to the tectal posterior boundary. Those intervals were defined by tracing ten concentric circles from the optic chiasm to the posterior boundary of the tectum, and tract widths were measured as the distance between the two outermost axons intersecting each circle. The width was normalized to the size of the brain measured from the optic chiasm to the posterior boundary of the tectum (Figure 3B). Quantification shows that the RGC axon pathway width of morphant embryos is similar to controls in the optic tract but is increased (by up to 35%, ∼40 μm) in the tectal region. This indicates that RGC axons of miR-182 morphants are appropriately bundled along the optic tract but that they project more expansively across the tectum compared to controls (Figure 3B), suggesting that miR-182 is involved in restricting the targeting area of RGC axons within the tectum. Though the described axon defect appears modest in terms of size, in comparison to the size, approximately 150 μm, of the tectal neuropil at this age, this 40 μm expansion of the projection in the target represents a significant change in retinotectal connectivity.

Because the blastomere microinjection approach targets the entire CNS, the axonal phenotype could be attributed to a loss of function of miR-182 in the RGCs (i.e., autonomous) or in the cells forming the pathway substrate in the brain (i.e., non-autonomous), although the latter possibility is rather unlikely due to the absence of miR-182 expression in the midbrain. To formally distinguish between these possibilities, we abolished miR-182 function specifically in retinal cells by electroporating miR-182 MO, plus a mCherry reporter, into stage 26 eye primordia when RGC axonogenesis is just beginning. The phenotype of miR-182 MO eye-electroporated embryos was similar to that of blastomere-injected miR-182 morphants, with both exhibiting an expanded RGC axon targeting area in the tectum (Figures 3C, 3D, and S3H). Finally, to validate the specificity of the miR-182 MO, we performed rescue experiments by electroporating retinal cells of stage 26 morphant embryos with miR-182 mimic or control mimic. The electroporation of miR-182 mimic, but not control mimic, induced a re-expression of miR-182 in retinal cells (Figure S3I) and rescued the guidance phenotype of miR-182-depleted RGC axons in the tectum (Figures 3E and 3F). This confirms that the phenotype observed in miR-182 morphants is due to the specific knockdown of this miRNA in retinal cells. Altogether, these data show that, in vivo, miR-182 acts cell autonomously in RGCs to delimit axons to a restricted area within the tectum.

### miR-182 Modulates RGC GC Responsiveness to Slit2

**Figure 4:**
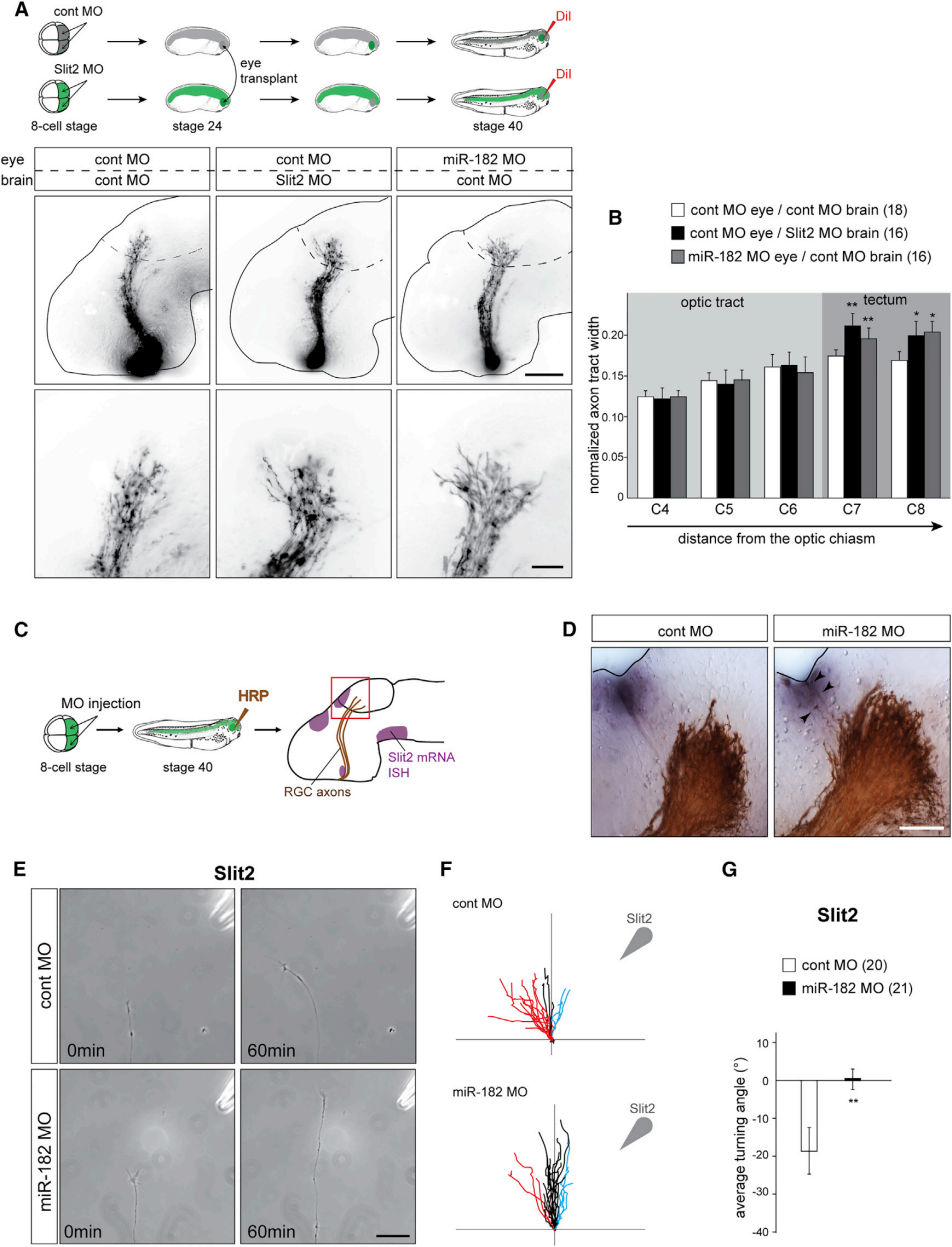
miR-182 Is Involved in Slit2-Driven RGC Axon Guidance and Targeting In Vivo and In Vitro (A) Schematic representation of the experimental protocol and representative images of brains, where RGC axons are stained with DiI. (B) Quantification of pathway width. Numbers of brains analyzed are between brackets. (C and D) Schematic representation of the experimental protocols (C) and representative images (D) of brains, where RGC axons are stained with HRP and Slit2 mRNAs are revealed by ISH. (E–G) In vitro turning assay on stage 35/36 RGC axons cultured for 24 hr and isolated from their cell bodies. (E) Representative images of control of miR-182 morphant RGC GC before and 60 min after being exposed to a gradient of Slit2 established from a pipette (top right corner) set at 45° angle from the initial direction of growth. (F) Tracings of RGC axons are analyzed. The source of the guidance cue is indicated by the arrowhead. Red, black, and blue traces represent, respectively, repulsive behaviors (angle < −5°), nonsignificant changes in the direction of growth (−5° < angle < 5°), and attractive turning (angle > 5°). (G) Quantification of the average turning angle. Numbers of GCs analyzed are between brackets. Values are mean ± SEM (B and G). Two-way ANOVA followed by Bonferroni post-test (B) or Mann-Whitney test (G), ^∗^p < 0.05, ^∗∗^p < 0.01. Cont, control; HRP, horseradish peroxidase; ISH, in situ hybridization; MO, morpholino oligomer; RGC, retinal ganglion cell. Scale bars, 150 μm (A, top panels), 50 μm (A, bottom panel, and D), and 30 μm (E). See also Figures S4 and S5.

The aberrant expansion of the projection observed in the miR-182 morphant tecta suggests that miR-182 may regulate the responsiveness of RGC axons to tectal repulsive cues that restrict the targeting area. Among multiple cues expressed within the tectum, the repulsive cue Slit2 is known to play a role in confining the growth of axons to specific areas (Erskine et al., 2000, Piper et al., 2006). Therefore, we hypothesized that Slit2 is involved in delimiting the RGC axon-recipient area of the tectum. To test this, we first asked whether loss of Slit2 in the brain causes a phenotype similar to that seen with miR-182 depletion. MO successfully blocked Slit2 translation (Figure S4). To achieve Slit2 knockdown in the brain, but not in the eye, control wild-type eyes were transplanted into Slit2 morphant host embryos at stage 24 and the RGC axon projections were subsequently assessed at stage 40 by DiI anterograde labeling (Figure 4A). In these embryos, RGC axons grow appropriately through the optic tract but project over a larger area in the tectum (Figures 4A and 4B), confirming the function of Slit2 as a target-restricting cue for RGC axons in vivo. This phenotype is similar to miR-182 morphant eye projections (Figure 3), consistent with the possibility that miR-182 interacts with Slit2 signaling in RGC axons. Moreover, covisualization of Slit2 (ISH) and RGC axons (horseradish peroxidase [HRP] anterograde labeling) at stage 40 shows that RGC axons grow closer to the Slit2-expressing tectal territory in miR-182 morphants than in control embryos, with some axons even invading Slit2 domains (Figures 4C and 4D). These results indicate that miR-182-depleted RGC axons fail to respond appropriately to Slit2 in vivo, resulting in targeting defects.

To test whether miR-182 alters axonal Slit2 sensitivity, we used the GC turning assay (Lohof et al., 1992). Stage 35/36 eye explants were cultured for 24 hr, a period that corresponds to the time when the RGC axons are beginning to enter the optic tectum in vivo (Piper et al., 2006). Turning assays were performed on axons severed from their cell bodies to exclude soma-derived effects. Control RGC axons showed robust repulsive turning from the Slit2 gradient (average turning angle of −18.7 ± 5.28°) (Figures 4E–4G) (Piper et al., 2006). By contrast, miR-182 morphant axons failed to exhibit a turning response to a Slit2 gradient (average turning angle of +1.91 ± 3.58°). These results show that Slit2-induced repulsive turning requires miR-182 activity and that this requirement is local. However, miR-182 morphant axons are still repelled by Sema3A, another guidance cue involved in target restriction in the tectum (Figures S5A–S5C). Thus, axonally localized miR-182 appears to regulate the responsiveness of GCs specifically to Slit2.

### miR-182 Regulates Slit2-Induced Cfl1 mRNA Translation

We next examined the mechanisms of action of miR-182 as a modulator of Slit2-induced axon guidance and targeting. Slit2-induced repulsive turning of RGC GCs is reported to be dependent upon LPS (Piper et al., 2006). Given the preceding findings, we reasoned that axonal miR-182 may mediate Slit2 signaling by targeting mRNAs that are locally translated in RGC GCs in response to Slit2.

**Figure 5:**
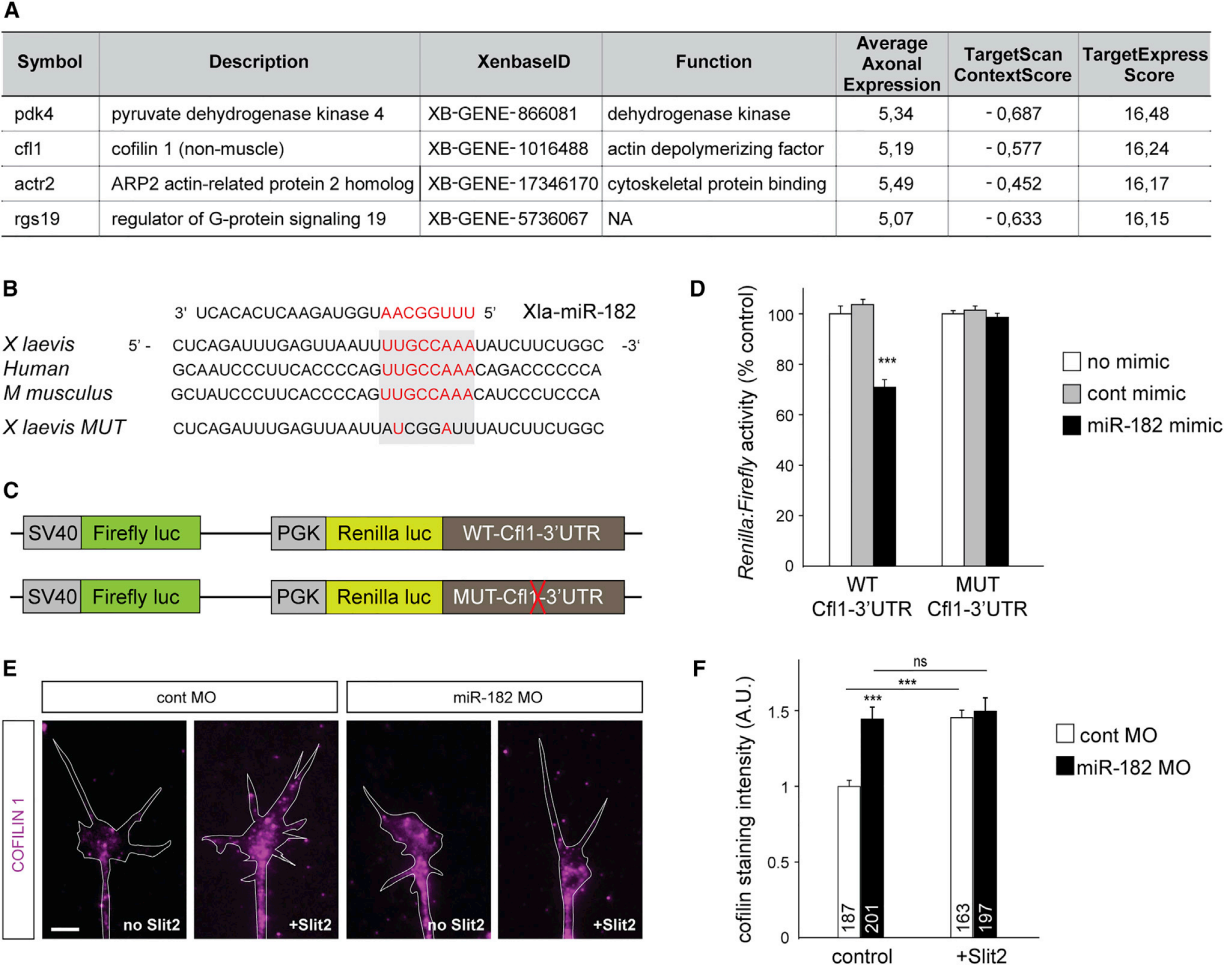
miR-182 Targets Cfl1 mRNA and Regulates Its Expression in RGC Axons (A) Top predicted miR-182 targets expressed in *Xenopus laevis* growth cones. (B) Sequence alignment of the 3′ UTR of Cfl1. The predicted miR-182 binding site is highlighted in red. (C) Schematic representation of *Xenopus* Cfl1-3′ UTR, subcloned downstream of a dual Renilla:Firefly luciferase reporter. (D) Quantification of reporter activity in HEK293T cells. (E and F) Representative images (E) and quantification (F) of Cfl1 immunostaining. White lines delineate RGC growth cones. Bath application of Slit2 was used at a suboptimal concentration to avoid collapse. Values are mean ± SEM (D and F). Numbers of GCs analyzed are indicated in bars (F). ANOVA followed by Bonferroni post-test, ^∗∗∗^p < 0.001. ns, nonsignificant; cfl1, Cfl1; cont, control; MO, morpholino oligomer; MUT, mutated; WT, wild-type. Scale bar, 5 μm (E). See also Table S2.

To gain insight about miR-182 putative targets in axons, we use our recently developed algorithm, TargetExpress (Ovando-Vázquez et al., 2016). We identified 1,064 potential miR-182 targets expressed in *Xenopus* RGC growth cones (Zivraj et al., 2010) (see Supplemental Experimental Procedures for details). Pyruvate dehydrogenase kinase 4, a metabolic enzyme with no known activity in axons and no known link to Slit2, has the highest probability and Cfl1 has the second-highest probability of miR-182 targeting (Figure 5A; Table S2). The 3′ UTR of Cfl1 mRNA is predicted to contain one highly conserved miR-182 8-mer binding site (Figure 5B). Slit2 induces the local synthesis of Cfl1, a regulator of actin cytoskeleton dynamics, in GCs, and this is known to mediate RGC GCs’ repulsive responses to Slit2 (Piper et al., 2006). We thus hypothesized that miR-182 modulates GC responsiveness to Slit2 by locally silencing Cfl1 mRNA translation.

To assess this, we first validated that *Xenopus laevis* Cfl1-3′ UTR is a bona fide target of miR-182 through a dual Renilla:Firefly luciferase reporter assay in HEK293T cells. Cfl1-3′ UTR was subcloned downstream of Renilla luciferase (Figure 5C). With this dual luciferase construct, the expression and activity of the Renilla luciferase depends on Cfl1-3′ UTR regulation, whereas the Firefly luciferase activity is independent. The dual luciferase reporter was transfected into HEK293T cells, along with miR-182 or control mimic, and the activity of both luciferases was measured. The expression of miR-182, but not the control mimic, induced a significant decrease in the Renilla/Firefly activity ratio (−28.8% ± 2.7%) (Figure 5D). However, the control miR-182 mimic had no significant effect on the Renilla/Firefly activity ratio when the predicted miR-182 site of Cfl1-3′ UTR was mutated (Figures 5B–5D). This assay showed that *Xenopus laevis* Cfl1 mRNA is directly targeted and silenced by miR-182 through its predicted binding site.

We next determined whether miR-182 directly regulates Cfl1 expression levels in RGC GCs. As a first approach, we measured by quantitative immunostaining the expression level of Cfl1 protein in RGC GCs of control or miR-182 morphants (Figures 5E and 5F). Under basal conditions, Cfl1 expression is significantly increased in miR-182 morphant GCs (+45% ± 7%), indicating that miR-182 represses Cfl1 mRNA in the absence of a stimulus, maintaining a dormant state. After stimulation by Slit2, Cfl1 levels significantly increase (+45.7% ± 5%) in control RGC GCs, as previously reported (Piper et al., 2006). In contrast, in the absence of miR-182, Slit2 stimulation does not induce any further increase of Cfl1 protein level in RGC GCs (Figures 5E and 5F). Our results thus further indicate that miR-182 is required to mediate a Slit2-induced increase of Cfl1 expression in the GC.

**Figure 6:**
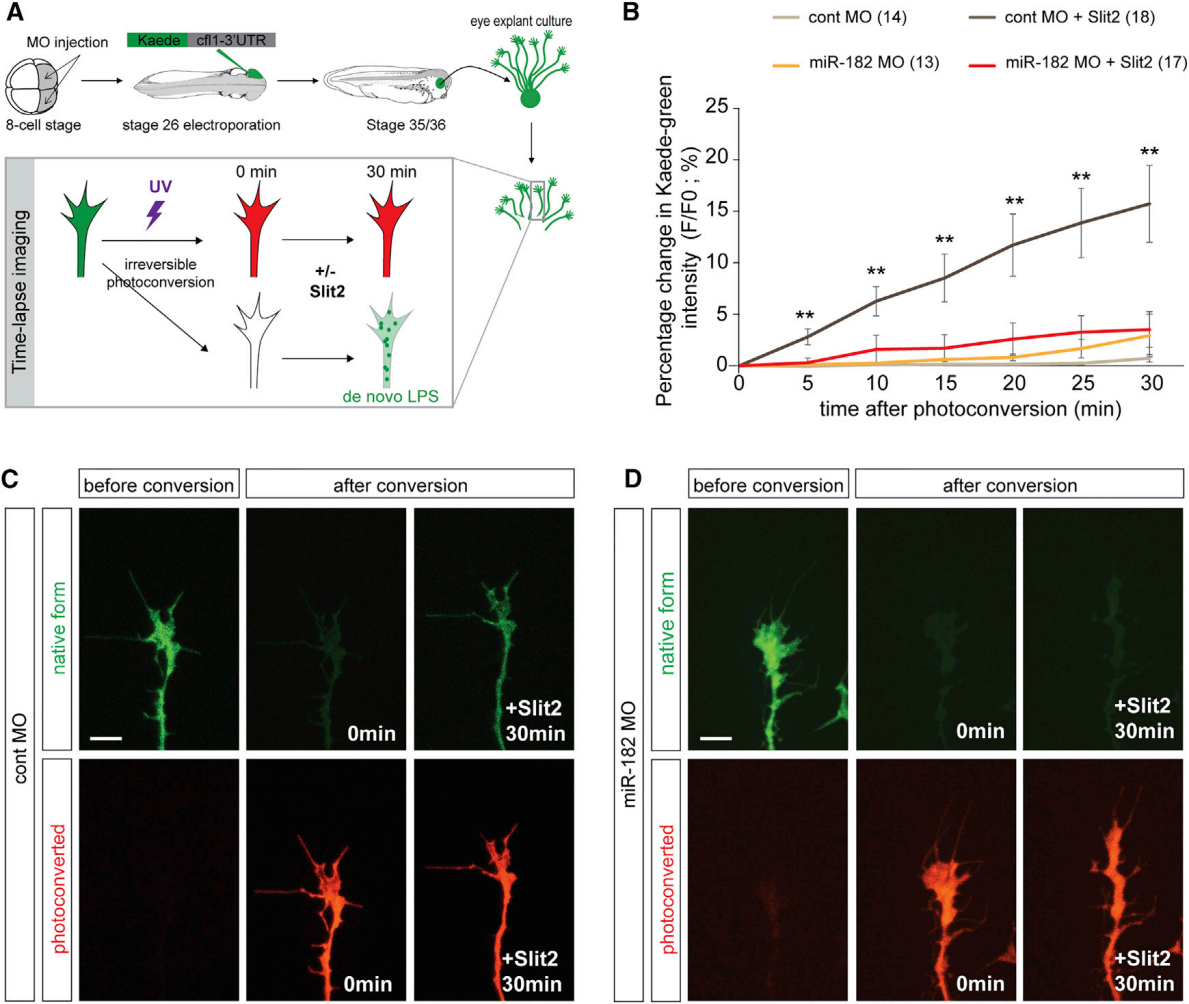
miR-182 Is Required for Slit2-Induced Local Translation of Cfl1 in RGC GCs (A) Schematic representation of the experimental protocol. After 24 hr, RGC axons were isolated from their cell bodies. Bath application of Slit2 at a suboptimal concentration was used to avoid collapse. Vehicle was used as control. Recovery of the newly synthesized Kaede green protein was monitored over time. (B) Quantification of the recovery of Kaede green signal. Data are presented as the percentage change of the fluorescence intensity (F) over time. Numbers of GCs analyzed are indicated in the legend of the graph. (C and D) Representative pre- and post-photoconversion images of severed control (C) or miR-182 morphant (D) axons. Values are mean ± SEM (B). Kruskal-Wallis test, ^∗^p < 0.05, ^∗∗^p < 0.01. Scale bars, 10 μm (C and D). Cont, control; LPS, local protein synthesis; MO, morpholino oligomer. See also Figure S6.

The increase of Cfl1 protein in the GC after Slit2 stimulation is consistent with de novo protein synthesis of Cfl1 in GCs. Alternatively, it may be due to increased transport of preexisting proteins from the axonal shaft. To distinguish between these possibilities, we tested directly whether miR-182 modulates Slit2-induced local de novo protein synthesis of Cfl1. To do so, a Kaede protein-based translation reporter (Leung and Holt, 2008) was generated to visualize live Cfl1 de novo protein synthesis in isolated GCs after Slit2 stimulation in vitro. The green fluorescence of native Kaede can be proteolytically and irreversibly photoconverted to red by UV illumination, and subsequent recovery of a green signal enables the detection of newly synthesized protein versus pre-existing protein. Because the miR-182 binding site is located in the Cfl1-3′ UTR, we made a reporter construct with the Kaede sequence linked to the 3′ UTR of Cfl1 mRNA (Kaede-Cfl1-3′ UTR). The Kaede-Cfl1-3′ UTR reporter construct was electroporated into the eye primordia of control or miR-182 morphant embryos at stage 26, and 12 hr later, eyes were explanted and grown for 24 hr in culture. To verify that the reappearance of the green signal was due to LPS specifically within the GC and not to transport from the cell body, axons were isolated from their cell bodies (Figure 6A). Under basal conditions, miR-182 morphant GCs exhibited a significantly higher basal level of Kaede fluorescence (+29% ± 9%) (Figure S6), consistent with our finding that miR-182 silences Cfl1 mRNA (Figure 5). For the green/red ratio comparative analysis, the intensity of the Kaede green signal was normalized to its intensity before photoconversion. In control GCs, the Kaede green signal reappears progressively after Slit2 stimulation (15.7% ± 3.7%, at 30 min), while no significant recovery is seen without stimulation (0.7% ± 0.3%, at 30 min). This confirms that Slit2 induces Cfl1 local translation directly in RGC GCs. In contrast, in the absence of axonal miR-182, no significant reappearance of the Kaede green signal is observed during the 30 min of imaging with or without stimulation by Slit2 (Figures 6B–6D), indicating that miR-182 is required to mediate Slit2-induced LPS of Cfl1 in RGC GCs in vitro.

Collectively, these results show that miR-182 modulates Cfl1 translation in RGC axons by both silencing Cfl1 mRNA under basal conditions and enabling its translation upon Slit2 stimulation.

### Slit2 Modulates miR-182 Activity in RGC GCs

**Figure 7:**
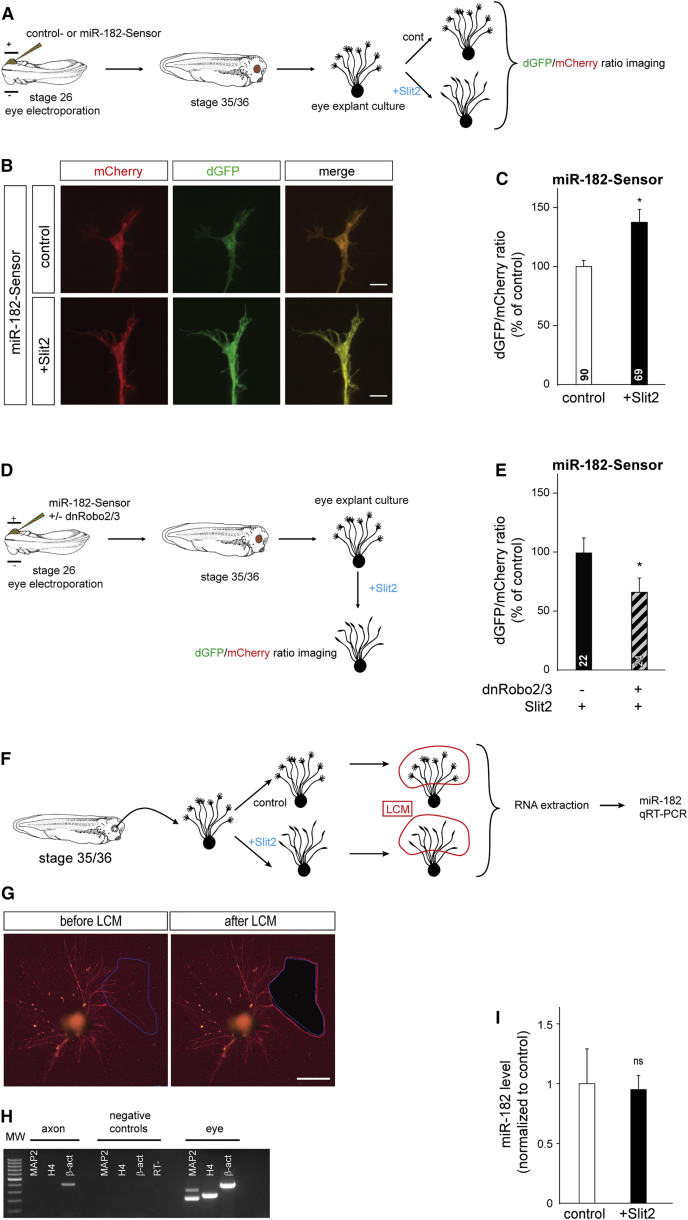
Slit2 Inhibits miR-182 Activity in RGC Axons without Decay (A, D, and F) Schematic representation of the experimental paradigm. Stage 35/36 retinal explants were cultured for 24 hr, and then Slit2 or vehicle were bath applied for 10 min. (B) Illustrative images of GCs from miR-182-Sensor-electroporated RGCs grown in culture. A clear example of dGFP/mCherry ratio increase is shown in (B). (C and E) Quantification of the dGFP/mCherry fluorescent ratio at the GC. (G) Illustrative images of explants and axons before and after LCM. (H) Illustrative gel of RT-PCR reaction for β-actin (β-act), MAP2, and histone H4 (H4) mRNA from cultured axons collected from stage 37/38 by LCM. In MAP2, H4, and β-act negative controls, PCR template was omitted. (I) Quantification of miR-182 by the ΔΔCt method in LCM axons. Values are mean ± SEM (C, E, and I). Mann-Whitney test, ^∗^p < 0.05. ns, nonsignificant; LCM, laser capture microdissection; RT−, RT no template negative control. Scale bars, 5 μm (B) and 200 μm (G). See also Figure S7.

The finding that miR-182 is a critical factor in Slit2 signaling pathway in the GC points to the possibility that Slit2 modulates miR-182 function in this neuronal compartment. To test whether Slit2 stimulation alters miR-182 activity directly in GCs, we electroporated the miR-182-Sensor or control-Sensor into eyes and made eye explant cultures (Figure 7A). Slit2 was bath applied to these cultures at a concentration determined to induce a protein synthesis-dependent response (Figure S7A). The fluorescence levels of dGFP and mCherry in RGC GCs were then measured. As expected, no change was detected in the dGFP/mCherry fluorescence ratio upon Slit2 stimulation in control-Sensor-expressing axons (+15% ± 10.5%) (Figures S7C and S7D). By contrast, a significant increase in the dGFP/mCherry ratio (+37.4% ± 10.8%) occurred upon Slit2 stimulation in the miR-182-Sensor-expressing axons (Figures 7B and 7C). Expression of the miR-182-Sensor or the control-Sensor did not affect Slit2-induced GC collapse, because the presence of either sensor does not alter GC responsiveness to Slit2 (Figure S7B). We further investigated whether and which Slit2 receptor variants, Robos, are putatively involved in Slit2-mediated miR-182 regulation. Robo2 and Robo3, but not Robo1, are expressed in *Xenopus* RGCs (Hocking et al., 2010, Piper et al., 2006). *Xenopus* Robo2 and Robo3 are, respectively, highly and poorly conserved with their rodent counterparts. While mammalian Robo3 silences Slit repulsion, non-mammalian Robo3 mediates it (Zelina et al., 2014). Using an experimental paradigm similar to that used earlier, we coelectroporated miR-182-Sensor with dominant-negative rat Robo2 (dnRobo2) and dominant-negative *Xenopus* Robo3 (dnRobo3) expression plasmids (Figure 7D). Dominant negatives have been previously used to assess the role of Robo signaling in axon guidance, including in *Xenopus* (Hocking et al., 2010). Fluorescence analysis shows that the dGFP/mCherry ratio is decreased in growth cones stimulated with Slit2 when dnRobo2/3 was electroporated compared to control (Figure 7E). Altogether, these data reveal that miR-182 is active and represses Cfl1 translation in the axonal compartment under basal conditions and that Slit2, via Robo2 and Robo3, inhibits its repressive activity in RGC GCs.

A common mechanism to modulate the activity of miRNAs is the regulation of their turnover or decay (Rüegger and Großhans, 2012). The Slit2-induced decrease in miR-182 activity in RGC GCs could thus arise due to the degradation of miR-182; alternatively, miR-182 could remain intact but be sequestered from its targets. To examine this, we asked whether miRNA levels changed in GCs following Slit2 stimulation by performing qRT-PCR for miR-182 on RGC axons. RGC axons were collected by LCM to avoid cell body contamination (Figures 7F and 7G), and the purity of the axonal material was confirmed by the presence of β-actin and the absence of dendritic marker MAP2 and nuclear marker histone H4 mRNA (Figure 7H). miR-182 levels were unaltered in Slit2-treated axons compared to controls, indicating that miR-182 is not degraded upon Slit2 signaling (−4.7% ± 10.9%) (Figure 7I). These results indicate that Slit2 triggers miR-182 inactivation in RGC GCs without causing its degradation and point toward the possibility of a reversible inactivation and activation mechanism.

## Discussion

During development, navigating GCs contain a rich transcriptome. Some of these transcripts are selected for translation to mediate cue-induced GC steering. However, the regulatory mechanisms conferring specificity of translation have remained largely elusive. We have addressed here whether miRNAs could contribute to the selection of specific transcripts for LPS in axon guidance. We show that elongating *Xenopus* RGC axons have a specific population of miRNAs and that miR-182 is enriched in this neuronal compartment. Our data show that miR-182 acts to modulate GC responsiveness to Slit2 in vitro and in vivo specifically within the tectum, where it plays a role in restricting axons to the appropriate target area. miR-182 does so, at least partly, by repressing the axonal translation of Cfl1, a key mediator of Slit2-induced GC repulsion. Slit2, in turn, triggers both a loss of activity of this miRNA, without leading to its degradation, and a concomitant rise in Cfl1 LPS. Collectively, these results indicate that the axon-enriched miR-182 is a key modulator of Slit2-mediated LPS during guidance.

To understand whether miRNAs could act as specific regulators of the axonal transcriptome, Next-Generation Sequencing-based profiling was first performed. Such a high-throughput unbiased approach has not been previously reported for axons. This revealed a complex repertoire of miRNAs within axons and GCs. Previous studies have documented not only the presence but also the enrichment and depletion of miRNAs in this neuronal compartment during development in various systems and organisms (Hancock et al., 2014, Natera-Naranjo et al., 2010, Sasaki et al., 2014), but the nature and abundance of miRNAs vary broadly among these studies, including ours. The differences could be attributed to variations in the types of cultures or methodologies or to bona fide biological differences. In support of this latter possibility, neurons of distinct types and stages express varied pools of axonal transcripts (Gumy et al., 2011, Zivraj et al., 2010). Some commonalities also appear. Rat superior cervical ganglia (Natera-Naranjo et al., 2010) and mouse cortical neurons (Sasaki et al., 2014) contain similar numbers of axonal miRNAs. In addition, miR-182 is enriched in mouse dorsal root ganglia distal axons (Hancock et al., 2014), and these cells respond to Slit2 (Nguyen-Ba-Charvet et al., 2001). This suggests that this miRNA might affect the axonal development in projection neurons regardless of cell type and species. It further indicates that miR-182 might have a conserved role in modulating cue-mediated axon guidance. Overall, it is tempting to speculate that each axon expresses a unique transcriptome and matching miRNome, depending on the cellular requirements at a given time of development, and that a limited set of conserved mRNA-miRNA pairs regulates key GC behaviors.

A key question is whether miR-182 acts locally to regulate protein synthesis. Evidence presented here indicates that miR-182 represses Slit2-induced Cfl1 protein synthesis specifically at the GC. First, miR-182 is present, abundant, and active in RGC axons and GCs, as shown by small RNA sequencing analysis, TaqMan PCR, in situ hybridization, and miRNA-Sensor-based detection approaches in unstimulated cultures. Its absence in RGC bodies by in situ analysis, together with its depletion in RGC bodies compared to other retinal cells revealed by TaqMan qPCR and the lack of miR-182-Sensor activity in RGC soma, further suggests that this miRNA is enriched in axons and GCs. miR-182 is thus likely to exclusively act in this compartment. Second, translational repression of Cfl1 by miR-182 appears to occur within GCs. In miR-182 morphants, Cfl1 protein immunoreactivity is increased specifically in this compartment, as detected by quantitative immunofluorescence. In addition, Cfl1-3′ UTR-driven expression of Kaede protein is higher in morphant GCs. The possibility that miRNAs regulate local translation was shown previously but not in the context of axon guidance. Several reports have documented that axonal miRNAs control levels of axonal protein (Aschrafi et al., 2008, Dajas-Bailador et al., 2012, Hancock et al., 2014, Kar et al., 2013, Wang et al., 2015, Zhang et al., 2013), including by modulating LPS of axonal transcript (Hancock et al., 2014, Wang et al., 2015). These previous reports were conducted in neuronal culture to investigate miRNA-regulated axon outgrowth. This study reveals that a miRNA modulates cue-induced LPS to promote GC steering during axon guidance. Along with the present dataset, these findings highlight the importance of miRNAs, as a class of molecule, in local regulation of translation within developing axons. What might be the added value for the GC of this miRNA-mediated LPS regulation? miRNAs could uniquely control the specificity of mRNA translation and contribute to selecting only a limited set of axonal targets for translation from the numerous pool of mRNAs present at the GC. In addition, miRNAs could limit, or avoid, unwanted expression of their mRNA targets outside the subregion of the GC close to cue exposure, thus enhancing precise spatial control of LPS. Finally, because miRNA action can be modulated, miRNAs may constitute an additional layer of regulation that could help set the specific time of LPS, avoiding spurious translation.

One finding is that Cfl1 LPS is not triggered by Slit2 exposure in miR-182 morphant axons, as shown by immunofluorescence and Kaede reporter construct. If miR-182 silences Cfl1 expression in the GC until a cue is encountered, Slit2-induced Cfl1 translation should occur even in the absence of miRNA. Several explanations can be provided for these results. First, the elevated levels of Cfl1 detected in miR-182 morphant axons may negatively feed back on Cfl1 LPS and prevent a further increase in Cfl1 levels. In the absence of miR-182, Cfl1 LPS would thus be uncoupled from Slit2 stimulation and Slit2 would be unable to affect the translational status of Cfl1 mRNA. Second, miR-182 loss of function may deregulate additional direct targets, other than Cfl1, implicated in the Slit2 signaling cascade or regulating LPS per se. In support of this, miR-182 is predicted to silence cofactors of mTOR, as well as mitogen-activated protein kinases (MAPKs) and associated or interacting proteins, all known to be important for Slit2-induced Cfl1 LPS (Piper et al., 2006). Furthermore, miR-182 is predicted to target a few transcripts involved in translation and known to be present in RGCs (Zivraj et al., 2010). Accordingly, miR-182 inactivation by Slit2 would impinge on multiple pathways that would converge to modulate Cfl1 LPS.

Although miRNAs were initially thought to be stable, the active degradation of mature miRNAs was recognized as a key process to modulate miRNA homeostasis (Rüegger and Großhans, 2012). This prompted us to investigate whether mature miR-182 levels decrease upon Slit2 exposure. However, we do not detect any change in miR-182 levels by qPCR. These results contrast with a report documenting that miR-182 decays in neurons within 90 min of stimulation (Krol et al., 2010a). Because this fast degradation was observed in mature neurons, but not in immature neurons (Krol et al., 2010a), this discrepancy may be explained by developing, and not fully differentiated, RGCs being used in the present work and/or by the varying type and length of stimulus exposure employed. However, our finding is in agreement with another study, which showed in dendritic spines that BDNF lifts the repression that miR-134 exerts on limk1 without altering the miRNA level (Schratt et al., 2006). From this emerges a putative common regulatory mechanism of miRNA inactivation in subregions of neurons not relying on degradation. The loss of activity of miR-182 without its associated decay might be induced by RBPs. RBPs are reported to compete with miRNAs for 3′ UTR binding regions or to bind directly to miRNAs, counteracting miRNA-mediated target repression. RBPs also cooperate with miRNAs to regulate mRNA silencing through shared mRNA *cis*-acting elements and/or through promoting and modulating RISC-mediated repression (Gardiner et al., 2015, Krol et al., 2010b). It is thus conceivable that Slit2 activates a competing RBP or inactivates a cooperating RBP, and this in turn terminates miR-182-mediated Cfl1 repression. One possible advantage of reducing miRNA activity without clearing it from neuronal compartments is that miRNAs can be readily available for future function without the costly need to transcribe and ship new molecules to regions far from the cell body. This type of reversible and bidirectional mechanism would be particularly well suited to these compartments, which are constantly exposed and respond rapidly to various stimuli.

In conclusion, we provide evidence demonstrating that a miRNA, miR-182, acts locally at the GC to confer selectivity of Slit2-induced Cfl1 translation, pointing to the following model. Under basal conditions, miR-182 keeps Cfl1 mRNA silent in RGC axons. Upon Slit2 stimulation, miR-182 activity is abolished in RGC GCs. This leads to the local de-repression of Cfl1 mRNA and its concomitant translation in the GC, while other mRNAs are kept silent by their own repressors. This localized burst of Cfl1 de novo synthesis, in turn, locally affects the cytoskeletal dynamics, subsequently inducing GC repulsive turning. Conceptually, different axonal miRNAs might silence different sets of mRNAs in the GC, preventing their LPS and constituting a reserve pool of mRNAs ready to be translated on demand. Inhibition of specific miRNA activity in the GC, in response to acute stimulation by guidance cues, will therefore act as a switch to relieve specific mRNAs from repression on site in the GC. Such a mechanism could represent an efficient way to ensure rapid selective translation, aiding the immediate response of the GC.

## Experimental Procedures

### Embryos

*Xenopus laevis* embryos were obtained by in vitro fertilization as previously described (Cornel and Holt, 1992), raised in 0.1× modified Barth’s saline at 14°C–22°C, and staged according to Nieuwkoop and Faber (1994). All animal experiments were approved by the University of Cambridge and University of Trento Ethical Review Committees.

DNA plasmids, antisense oligonucleotides, and mimics used are described in Supplemental Experimental Procedures.

### Blastomere Microinjection

A total of 5 ng of morpholinos were injected into both dorsal animal blastomeres of eight-cell-stage embryos as described previously (Piper et al., 2008).

### Electroporation

DNA constructs, morpholinos, or miR-182 mimics were electroporated in one eye of stage 26 embryos, with conditions similar to those previously described (Falk et al., 2007).

### Optic Pathway Analysis

Stage 40 embryos were anesthetized and fixed in 4% paraformaldehyde (PFA) for 2 hr to overnight. RGC axons were labeled by anterograde DiI filling of the eye or directly visualized by mCherry fluorescence when electroporated. Brains were dissected and mounted to visualize the optic tract on the contralateral side of the brain. The *z* stacks of serial images comprising the entire contralateral optic pathway were captured. Analysis on the width and the length of the pathway were performed as previously described (Walz et al., 2002), except that all measurements were normalized to brain size.

### Retinal Explant Culture

Whole retinas of anesthetized stage 35/36 or 37/38 embryos were dissected and cultured at 20°C for 24 hr, unless otherwise stated, in 60% L15 minimal medium (Invitrogen) and 1× penicillin, streptomycin, and fungizone on glass coverslips (Bellco) or glass-bottom dishes (MatTek) coated with poly-L-lysine (10 μg/mL, Sigma) and laminin (10 μg/mL, Sigma).

### Axonal Small RNA Sequencing

For 48 hr, 1,000 whole eye explants from stage 37/38 were cultured. Eye explants and contaminating cells were manually removed to isolate distal axons only. Total RNA was extracted from both the axonal and the explant fractions by phenol-chloroform extraction. The quality, quantity, and purity of the axonal material were tested as described in Supplemental Experimental Procedures. Small RNA libraries were prepared without pre-amplification, using the TruSeq Small RNA Library Preparation Kit (Illumina) and sequenced on a MiSeq sequencer (Illumina). Sequencing data analysis was performed as described in Supplemental Experimental Procedures.

### Laser Capture Microdissection

LCM of axons and RGC soma were performed on LMD6500 (Leica). The quality, quantity, and purity of the collected RNA were assessed as described in Supplemental Experimental Procedures

#### Axons

Stage 35/36 whole eye explants were cultured on RNase-DNase free polyester (POL) membranes (Leica) for 24 hr and then processed for LCM as previously described (Zivraj et al., 2010), except that 1% PFA was used instead. Distal axons and explants from the same culture were collected in separate tubes. RNA was extracted using the RNAqueous-Micro kit (Ambion). In vivo, laser capture of axons was performed from stage 40 sections, and RNA was extracted using the Single Cell kit (Norgen).

#### RGC Soma

LCM of the RGC layer was performed on sectioned stage 40 embryos, and stage 37/38 whole eyes were used as control. RNA was extracted using the Total RNA Purification Kit (Norgen).

### TaqMan qPCR for miR-182

Total RNA collected following LCM (described earlier) was retro-transcribed using the TaqMan MiRNA Reverse Transcription Kit. The cDNA obtained was used for the TaqMan Micro RNA assay using xtr-miR-182-5p and U6 snRNA-specific primers and probes and the TaqMan Universal Master Mix II (MMIX II) no AmpErase Uracil N-Glycosylase (UNG) (all Thermo Fisher). Reactions were run on a Bio-Rad CFX96 Real-Time System. For quantitative analysis, cycle threshold (Ct) mean values were measured in biological triplicates or more, and the ΔΔCt method (Schmittgen and Livak, 2008) was applied as follows: fold change is −1/(2ˆ[(Ct_miR-182_ − Ct_U6_)_RGC_ − (Ct_miR-182_ − Ct_U6_)_eye_]).

### Quantitative Fluorescence Analysis

#### Quantitative Fluorescence of RGC GCs

Isolated GCs were selected at random with phase optics. To avoid subjective bias, analyses were performed blind to the experimental condition. For each experiment, all acquisitions were performed during the same day with the same settings. The outline of each unsaturated GC was traced to define a region of interest (ROI), and the mean intensity of each channel was measured using ImageJ or Leica Application SuiteX software. The background fluorescence was measured in a ROI as close as possible to the GC selected and subtracted to the GC mean fluorescence value.

#### Quantitative Fluorescence of Retinal Cells

Quantitation on cryosectioned retina pictures was performed as described earlier, except that retinal cells in the photoreceptor (PR) layer and the innermost part of the inner nuclear layer were defined as the ROI.

### GC Turning Assay

Turning assays were performed as described in Campbell and Holt (2001). Further details are provided in Supplemental Experimental Procedures.

### miRNA In Situ Hybridization

miRNA ISH protocols for (1) whole-mount, (2) cultured GCs, and (3) for retinal sections were adapted from (1) Wienholds et al. (2005), (2) Han et al., 2011, and from (3) Baudet et al. (2011) and Obernosterer et al. (2007). More details are provided in Supplemental Experimental Procedures.

### HRP Axon Tracing

HRP axon tracing and Slit2 ISH were performed as in Piper et al. (2006) on stage 40 embryos. An overview of the HRP labeling protocol is available in Supplemental Experimental Procedures.

### Dual Luciferase Reporter Assay

Using Jet prime reagent (Polyplus Transfection), 250 ng of psiCHECK2-Cfl1-WT-3′ UTR or psiCHECK2-Cfl1-MUT-3′ UTR were transfected with or without 12 pmol of control mimic or miR-182 mimic into HEK293T cells plated 12 hr earlier on 48-well plates. The activity of both Renilla and Firefly luciferase was measured 36 hr after transfection using the Dual Luciferase Reporter Kit (Promega) and a DLReady TD-20/20 single-tube luminometer (Turner Biosystems).

### Live Imaging of the Kaede-Cfl1-3′ UTR Translation Reporter in Cultured Axons

After injection of control MO or miR-182 MO at the eight-cell stage, one eye of the embryo was electroporated at stage 26 with pCS2+Kaede or pCS2+Kaede-Cfl1-3′ UTR reporter constructs. Electroporated eyes were dissected at stage 36 and cultured for 24 hr to allow axonal growth. Before cue stimulation, RGC axons were isolated from their cell bodies by manual removal of the explant. Analysis of local translation of the Kaede reporter was performed as previously described for the β-actin-3′ UTR (Piper et al., 2006, Piper et al., 2008). A brief description is available in Supplemental Experimental Procedures.

### Statistical Analysis

Each experiment was conducted at least three times unless otherwise stated. For all tests, the significance level was α = 0.05. Data were analyzed with Prism 5 (GraphPad). The normal distribution of datasets was tested by the D’Agostino and Pearson omnibus normality test. Statistical tests used are mentioned in figure legends.

## Author Contributions

Conceptualization and Writing—Original Draft, A.B., C.E.H., and M.-L.B.; Investigation and Validation, A.B., A.I., S.B., F.L., C.O.-V., E.C., S.L., M.R., S.St., S.N., P.S., C.A.-G., and M.-L.B; Software, C.O.-V., S.St., and C.A.-G; Resources, E.M.; Supervision, A.B., E.M., C.A.-G., C.E.H., and M.-L.B.

## Accession Numbers

The accession number for all the RNA-seq data reported in this paper is GEO: GSE86883.

## Supplemental Information

Document S1. Supplemental Experimental Procedures and Figures S1–S7

Table S1. RGC Axons miRNA Profiling Data, Related to Figure 1 Full list of mature miRNAs detected in RGC axons with small RNA sequencing. The most abundant ones are presented in Figure 1.

Table S2. Predicted miR-182 Targets Expressed in *Xenopus laevis* Growth Cones at Stage 32, Related to Figure 5 Full list of predicted miR-182 targets expressed in stage 32 cultured RGC growth cones. The growth cone transcripts with the highest probability of targeting are presented in Figure 5.

Document S2. Article plus Supplemental Information
