# Supplementary material for: miR-182 Regulates Slit2-Mediated Axon Guidance by Modulating the Local Translation of a Specific mRNA
Source: Cell Rep. 2017 Jan 31;18(5):1171–86. doi: 10.1016/j.celrep.2016.12.093 (PMC5300892; doi:10.1016/j.celrep.2016.12.093)
Supplement: Document S1. Supplemental Experimental Procedures and Figures S1–S7 [file mmc1.pdf]

Cell Reports, Volume *18*

Supplemental Information

miR-182 Regulates Slit2-Mediated Axon Guidance by Modulating the Local Translation of a Specific mRNA

Anaïs Bellon, Archana Iyer, Simone Bridi, Flora C.Y. Lee, Cesaré Ovando-Vázquez, Eloina Corradi, Sara Longhi, Michela Roccuzzo, Stephanie Strohbuecker, Sindhu Naik, Peter Sarkies, Eric Miska, Cei Abreu-Goodger, Christine E. Holt, and Marie-Laure Baudet

Supplemental Figures

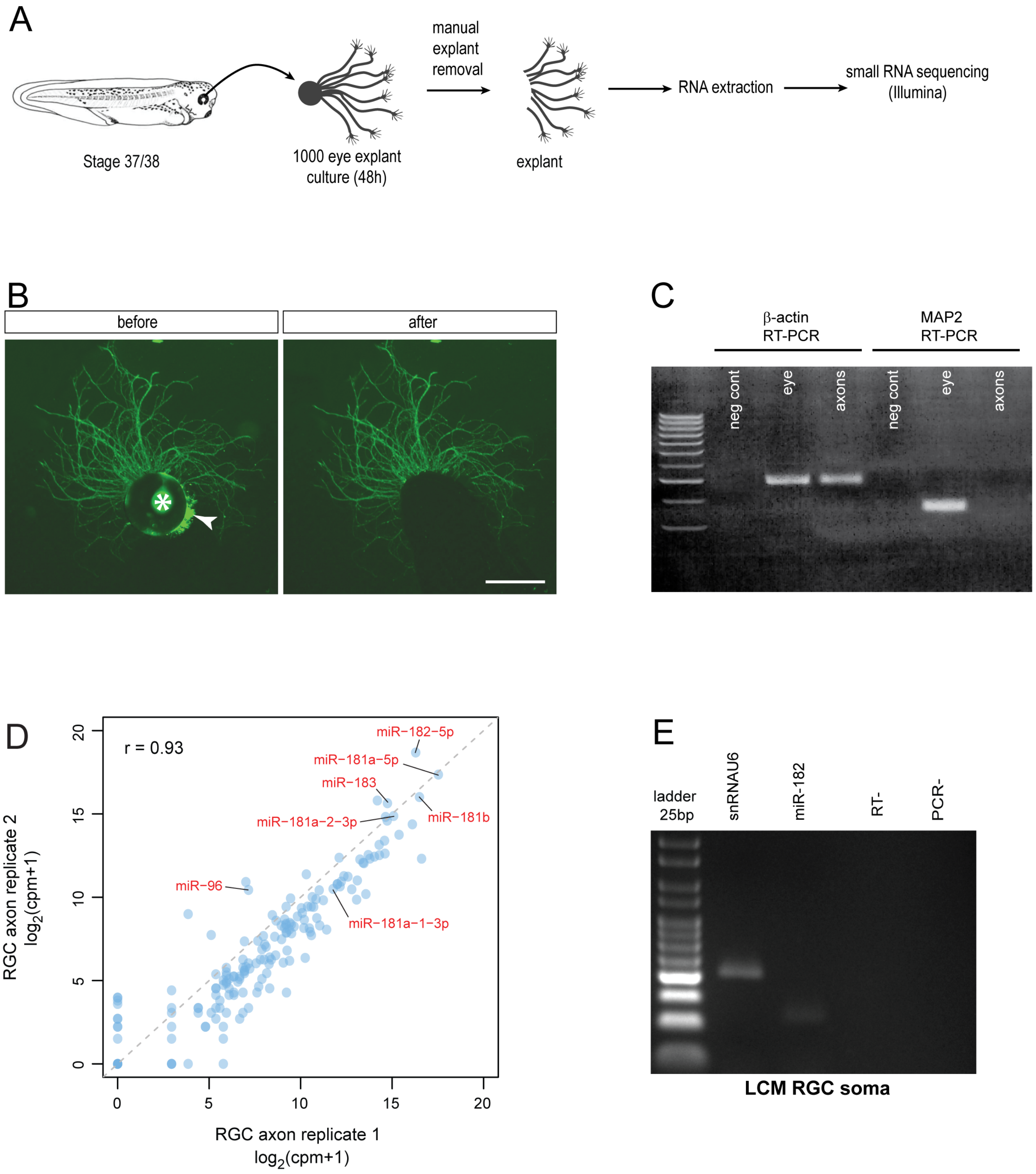

Figure S1: Validation of axonal RNA purification and sequencing, Related to Figure 1.
(A) Schematic representation of the experimental protocol. 1000 stage 37/38 eye explants were cultured. After 48h, explants were manually removed, and total RNA was extracted from the axonal fraction thus obtained. Small RNA cDNA libraries were generated and sequenced. **(B)** Representative images of 48h cultured stage 37/38 eye explant, before (left) and after (right) removal of the eye (star) and contaminating cells (arrowhead). Scale bar 300µm. **(C)** RT-PCR for MAP2 and β-actin mRNA performed on RNA extracted from the axonal or eye fraction. Both β-actin and MAP2 mRNA are detected in the eye fraction while only β-actin mRNA is detected in the axonal one, suggesting that the RNA extracted from the axonal fraction after removal of the explant is devoid of RNA contamination from cell bodies and dendrites. **(D)** Pearson correlation analysis of sequencing reads of axonal miRNAs between the two replicates. Values on axes show log2(cpm+1) for each sample. The Pearson correlation coefficient of 0.93 reported on top of the plot indicates a good correlation between the two replicates. **(E)** Illustrative gel showing TaqMan qPCR performed on RNA extracted from laser captured RGC layer detecting traces of miR-182 in RGC soma. RNA-U6 was used as a positive control. RT-, RT no template negative control; PCR-, PCR no template negative control.

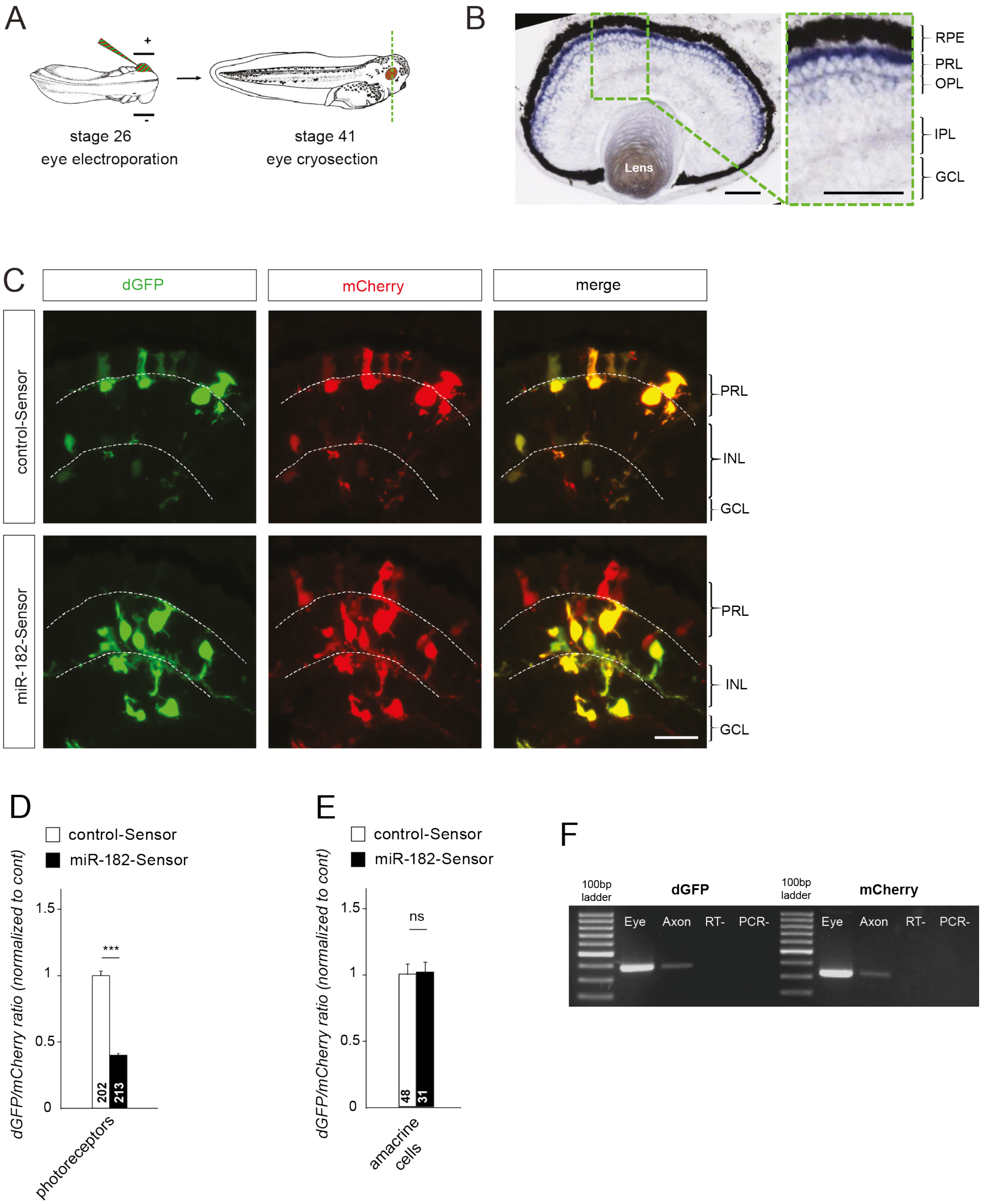
A
stage 26
eye electroporation
stage 41
eye cryosection
B
Lens
RPE
PRL
OPL
IPL
GCL
C
dGFP
mCherry
merge
control-Sensor
miR-182-Sensor
PRL
INL
GCL
D
control-Sensor
miR-182-Sensor
dGFP/mCherry ratio (normalized to cont)
***
202
213
photoreceptors
E
control-Sensor
miR-182-Sensor
ns
48
31
amacrine cells
F
100bp ladder
dGFP
100bp ladder
mCherry
Eye
Axon
RT-
PCR-

Figure S2: The activity of miR-182-Sensor inversely correlates with the expression of miR-182 in retinal cells, Related to Figure 2.

(A) Schematic representation of the experimental protocol. miR-182- or control-Sensor (shown in Fig. 2A) were electroporated in one eye at stage 26. Broken green line indicates the plane of section for the images shown in (B). **(B)** Representative images of endogenous miR-182 distribution detected by in situ hybridization on stage 40 retinal section. **(C)** Illustrative images of retina electroporated by control- or miR-182-Sensor. **(D-E)** Quantification of dGFP / mCherry fluorescence ratio in photoreceptor and amacrine cells following electroporation of control- or miR-182-Sensor. Values are mean ± SEM. Mann Whitney test. ***$p < 0.001$. ns, nonsignificant. 415 photoreceptor cells and 79 amacrine-like cells (indicated in bars) were analyzed from three retinas per condition in total. **(F)** Illustrative gel following RT-PCR of dGFP and mCherry mRNAs on electroporated eye, collected at stage 37/38, and RGC axon collected by LCM from stage 40. To avoid detecting the parent plasmid, DNaseI treatment was carried out and RT was performed using oligo(dT). Presence of these two transcripts in axons indicates that Sensor construct is suitable to assess local activity of miR-182 in axons. GCL, ganglion cell layer; INL, inner nuclear layer; IPL, inner plexiform layer; OPL, outer plexiform layer; PRL, photoreceptor layer; RPE, retinal pigmented epithelium; RT-, RT no template negative control; PCR-, PCR no template negative control. Scale bars, 50µm (B), 25µm (C).

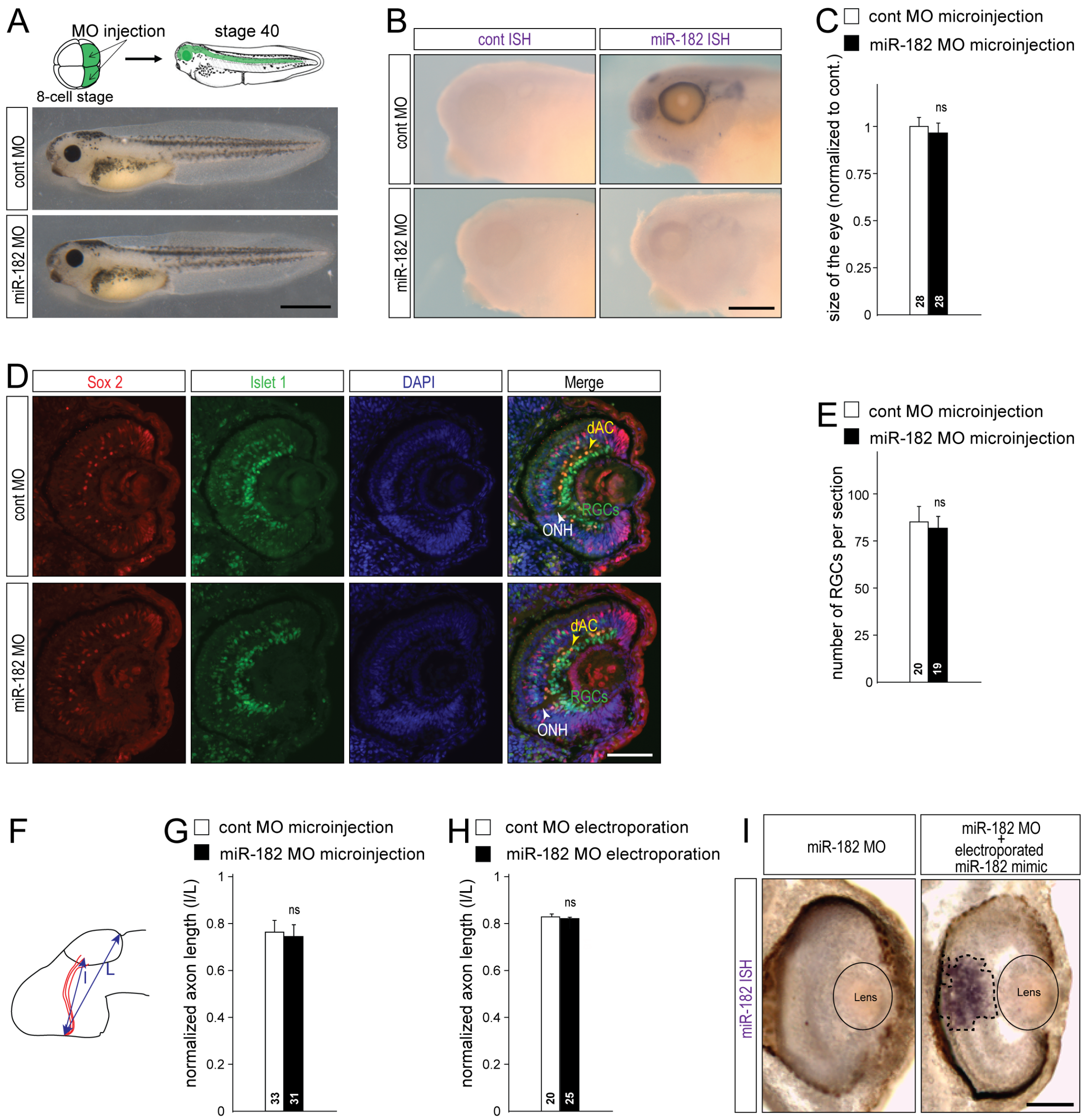
A
MO injection
stage 40
8-cell stage
cont MO
miR-182 MO
B
cont ISH
miR-182 ISH
cont MO
miR-182 MO
C
cont MO microinjection
miR-182 MO microinjection
size of the eye (normalized to cont.)
ns
1
0.75
0.5
0.25
0
28
28
D
Sox 2
Islet 1
DAPI
Merge
cont MO
miR-182 MO
dAC
RGCs
ONH
E
cont MO microinjection
miR-182 MO microinjection
number of RGCs per section
100
75
50
25
0
ns
20
19
F
l
L
G
cont MO microinjection
miR-182 MO microinjection
normalized axon length (l/L)
1
0.8
0.6
0.4
0.2
0
ns
33
31
H
cont MO electroporation
miR-182 MO electroporation
normalized axon length (l/L)
1
0.8
0.6
0.4
0.2
0
ns
20
25
I
miR-182 MO
miR-182 MO
+
electroporated
miR-182 mimic
miR-182 ISH
Lens
Lens

Figure S3: miR-182 MO efficiently knocks down endogenous miR-182 without affecting the generation of RGCs nor the growth of their axons, and miR-182 mimics rescues miR-182 expression, Related to Figure 3.

(A-E) Effect of the injection of control or miR-182 MOs in both dorsal blastomeres at eight cell stage embryos, driving the expression of the morpholino in the central nervous system, in *Xenopus laevis* embryos at stage 37/38 and 40. **(A)** Schematic of the experimental protocol and representative images of control and miR-182-injected embryos at stage 40. No major difference was observed between controls and miR-182 morphants morphology. **(B)** Representative images of ISH signal for control or miR-182 probes on stage 37/38 control or miR-182 morphant embryos. Most of the ISH signal for miR-182 in neural cells disappears in presence of miR-182 MOs, suggesting that miR-182 MOs efficiently knockdown endogenous miR-182. **(C)** Quantification of the size of the eye of control and miR-182 morphant embryos at stage 40 shows no significant differences between the two populations. **(D)** Representative images of cryosections of the eye of control or miR-182 morphant embryos at stage 40, at the level of the optic nerve head, after immunolabeling for Sox2 (red) and Islet1 (green) and counterstaining with DAPI (blue). **(E)** Quantification of the number of RGCs, per section, in stage 40 control or miR-182 morphant embryos. No significant difference was observed. **(F)** Schematic representation of the quantification method after injection in dorsal blastomeres or electroporation in the eye of control or miR-182 MOs, and DiI labeling of the RGC axons. The length of axons was assessed by measuring the distance from the optic chiasm to the longest RGC axons (l) normalized to the size of the brain from the optic chiasm to the posterior boundary of the tectum (L). **(G-H)** Graph showing the quantification of RGC axon length in control and miR-182 MOs injected **(G)** or electroporated **(H)** embryos. No significant difference in RGC axon length was detected in absence of miR-182. (**I**) Representative images of ISH for miR-182 on vibratome section of stage 37/38 morphant embryos after eye electroporation of control- or miR-182 mimics in the eye. Exogenous miR-182 mimics, but not control mimics, were detected in electroporated retinal areas (delineated by a dashed black line) in a miR-182 morphant background. Through the figure: values are mean ± SEM. Numbers of eye/embryo (C), retinal sections (E) and brains (G, H) analyzed are indicated within bars. All samples passed D'Agostino & Pearson Omnibus normality test. Student's t-test. ns, nonsignificant Scale bars, 1mm (A), 250µm (B), 100µm (D,I).

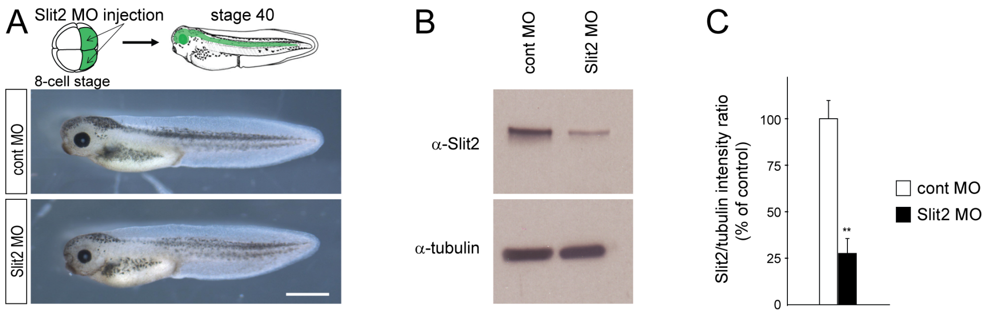

Figure S4: MO-mediated Slit2 knockdown affects the size of the eye, Related to Figure 4.
(A) Representative images of stage 40 embryos whose dorsal blastomeres have been injected with control or Slit2 MOs at 8 cell stage. **(B-C)** The efficiency of Slit2 knockdown was assessed by Western Blot. **(B)** Representative western blot for Slit2 and α-tubulin of head lysates from stage 40 control or Slit2 morphants. **(C)** Quantification of Slit2/ α-tubulin intensity ratio (n=3) shows a drastic reduction of Slit2 expression in the presence of Slit2 MO in the nervous system. Values are mean ± SEM. Wilcoxon paired test, **p<0.01. ns, nonsignificant; cont, control; MO, morpholino. Scale bar, 1mm (A).

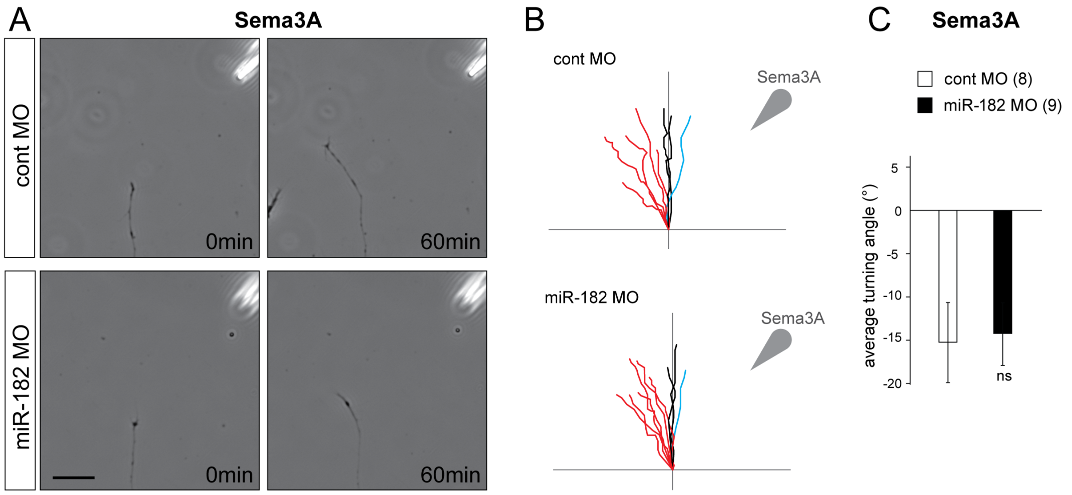

Figure S5: miR-182 is not required for Sema3A-induced repulsive turning of RGC axons in vitro, Related to Figure 4.

In vitro turning assay on stage 35/36 RGC axons cultured for 24h and isolated from their cell bodies. **(A)** Representative images of control or miR-182 morphants RGC GC before and 60 minutes after being exposed to a gradient of Sema3A established from a pipette (top right corner) set at 45° angle from the initial direction of growth. Scale bar, 30µm**.** **(B)** Tracings of RGC axons analyzed. Source of guidance cue is indicated by arrowhead. Red, black and blue traces represent, respectively repulsive behaviors (angle <-5°), nonsignificant changes in the direction of growth (-5°<angle<5°), and attractive turning (angle >5°). **(C)** Quantification of the average turning angle ± SEM. Numbers of GCs analyzed are between brackets. Mann-Whitney test, **p<0.01. ns, nonsignificant.

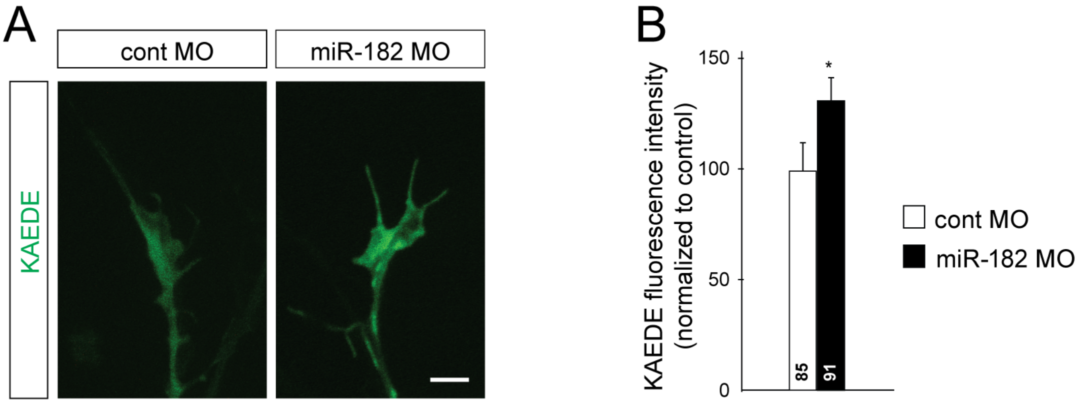

Figure S6: Basal expression level of Kaede-cofilin1 construct in RGC growth cones is regulated by miR-182, Related to Figure 6.
Comparison of the basal expression levels of Kaede-cofilin1 in control or miR-182 morphant RGC growth cones in vitro. **(A)** Representative images of control or miR-182 morphant Kaede-cofilin1 expressing growth cones. Scale bar, 10µm. **(B)** Quantification of Kaede fluorescent signal in control or miR-182 morphant growth cones. miR-182 knockdown increases the basal expression level of Kaede-cofilin1 in RGC growth cones. Values are mean ± SEM. Numbers of growth cones analyzed are indicated within bars. Unpaired Student's t-test, *p<0.05.

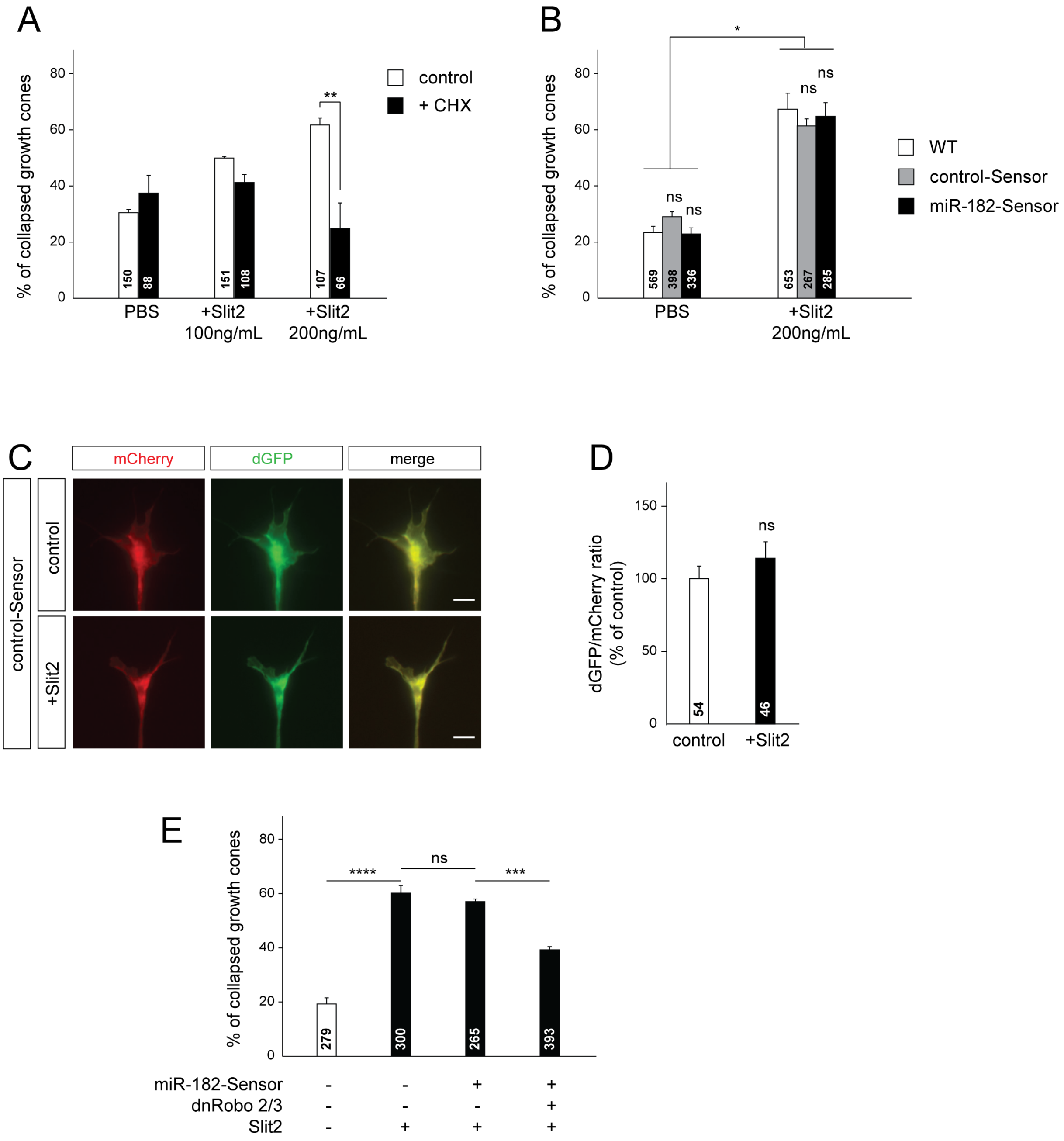

A
% of collapsed growth cones
0
20
40
60
80
control
+ CHX
**
150
88
151
108
107
66
PBS
+Slit2 100ng/mL
+Slit2 200ng/mL
B
*
ns
ns
ns
ns
WT
control-Sensor
miR-182-Sensor
569
398
336
653
267
285
PBS
+Slit2 200ng/mL
C
mCherry
dGFP
merge
control-Sensor
control
+Slit2
D
dGFP/mCherry ratio (% of control)
0
50
100
150
ns
54
46
control
+Slit2
E
****
ns
***
279
300
265
393
miR-182-Sensor - - + +
dnRobo 2/3 - - - +
Slit2 - + + +

Figure S7: Protein synthesis-dependent Slit2-induced collapse is not affected by the expression of miR-182 activity Sensor, Related to Figure 7.

(A) Quantification of collapsed growth cones from stage 35/36 retinal explants cultured for 24h following 10 min 100ng/mL or 200ng/mL Slit2 stimulation by bath application in the presence or absence of the translational blocker cycloheximide (CHX). At 200ng/mL a Slit2-induced PS-dependent collapse was observed. **(B)** Quantification of collapsed growth cones from stage 35/36 retina from wildtype embryos or embryos electroporated at stage 26 with control or miR-182-Sensor (shown in Fig. 2A), cultured for 24h and stimulated for 10 min by 200ng/mL Slit2 or PBS control. (**C**) Representative images of GCs following retinal electroporation of control-Sensor. (**D**) Quantification of dGFP / mCherry fluorescent ratio directly at the GC following 10 min Slit2 or PBS control bath application. Mann Whitney Test. (**E**) Quantification of collapsed growth cones from stage 35/36 retina from wildtype embryos or embryos electroporated at stage 26 with miR-182-Sensor and/or pCS2-dnRobo2-MT and pCS2-dnRobo3-MT, cultured for 24h and stimulated for 10 mins by 200ng/mL Slit2 or PBS control. Collapse was significantly reduced by 18% following electroporation of dnRobo2/3. Residual collapsed growth cones might be due to the fact that not all RGCs are targeted by electroporation. Values are mean ± SEM. Numbers of growth cones analyzed are shown within bars. Two-way ANOVA followed by Sidak multiple comparison test (A,B). $^{*}p<0.05$; $^{**}p<0.01$. (B) Not displayed on graph: WT, $^{****}p < 0.0001$; control-Sensor, $^{*}p<0.05$; miR-182-Sensor, $^{**}p < 0.01$. One-way ANOVA followed by Sidak multiple comparison post hoc test (E). $^{***}p< 0.001$; $^{****}p < 0.0001$. ns, nonsignificant. Scale bar, 5µm.

Table S1: RGC axon miRNA profiling data, Related to Figure 1**.**

The table presents the sequencing data of mature miRNAs from axonal sRNA-Seq libraries prepared from two independent axonal preparation of stage 37/38 retinal cultures (sample 1 and 2). For each library, the counts-per-million (cpm) were calculated by dividing the number of reads mapping to each miRNA (column 2 and 3) by the total reads from that library and multiplying by 1 million. For display purposes a count of 1 was added to each cpm, log2-transformed, and the mean was then calculated for each miRNA (column 4). The relative abundance in percentage was also calculated (column 5-7). The table is sorted by decreasing axonal cpm values.

Table S2: Predicted miR-182 targets expressed in *Xenopus laevis* growth cones at stage 32, Related to Figure 5.

The table presents predicted miR-182 targets expressed in stage 32 growth cones grown in culture for 24h (equivalent to in vivo stage 37/38) ranked by their TargetExpress scores (column 9). The TargetExpress score is based on the expression level for each gene (column 6) and their respective TargetScan Context+ score (column 7) for miR-182-5p. Average expression levels for each gene in stage 32 growth cones were obtained after vsnrma normalization of *Xenopus laevis* growth cone microarray data (GEO: GSE25166). Only genes with assigned GO terms and represented on the Affymetrix *Xenopus laevis* Genome Array are displayed.

Supplemental Experimental Procedures

Antisense oligonucleotides and mimics. FITC labeled and/or unlabeled antisense oligo morpholinos (MO) for xtr-miR-182 (5′-TGTGAGTTCTACCATTGCCAAA-3′), xla-Slit2-S (5’-ACTGAGGTCACTGTGGATAAAAAGA -3’, xla-Slit2-L (5’-TGACTGCTGACCTGAAAATTAAAGT-3’), control (5′-GTGTAACACGTCTATACGCCCA-3′) and standard control MOs (5′-CCTCTTACCTCAGTTACAATTTATA-3′) were obtained from Gene Tools. miR-182 mimics (miRIDIAN mimic 5’-UUUGGCAAUGGUAGAACUCACA-3’) and control mimics (miRIDIAN mimic negative control #1, 5′-UCACAACCUCCUAGAAAGAGUAGA-3′) were purchased from GE healthcare-Dharmacon.

DNA plasmids. miR-182-Sensor was generated as follows. d2GFP (destabilized GFP) from pd2EGFP-N1 vector (ClonTech) was subcloned into a pCS2+ vector, downstream of a CMV promoter. The first 150 nt of the 3’UTR of the *Caenorhabditis elegans* myosin heavy chain (unc54) gene (J01050.1), known to have no vertebrate miRNA binding sites (De Pietri Tonelli et al., 2006), and three fully complementary sequences to the *Xenopus laevis* miR-182, separated by 8 nt spacer, were then placed 3’ of the d2GFP (GenScript, Hong Kong). Finally, CMV-mCherry-SV40 was subcloned from a pCS2-mCherry parent vector into the pCS2-d2GFP-miR-182 plasmid to generate miR182-Sensor. The corresponding negative control plasmids, control-Sensor, was generated by inserting three tandem cassettes with an arbitrary sequence not complementary to any known miRNA (De Pietri Tonelli et al., 2006) instead of the three complementary sequences to miR-182.
pCS2+ Kaede-cofilin1-3′UTR reporter was generated by cloning CoralHue^{TM} Kaede (Bam HI/PstI blunt) into pCS2+ plasmid (BamHI/StuI). The 3’UTR of *Xenopus laevis* cofilin1 was amplified by PCR. The amplified fragment was cloned in the KpnI/XhoI sites of pCS2+Kaede.
psiCHECK2-cofilin1-WT-3’UTR luciferase construct was generated by extraction of cofilin1-3’UTR from pCS2+ Kaede-cofilin1-3′UTR by XhoI/KpnI and insertion into the multiple cloning site of the Renilla: firefly luciferase-expressing vector psiCHECK2 (Promega). psiCHECK2-cofilin1-MUT-3’UTR was generated by site-directed mutagenesis using QuickChange II XL kit (Agilent Technologies) and the following mutagenic primers containing miR-182 binding site mutations: 5'-GGATGGGTAAGACGCCAGAAGATAAATCCGATAATTAACTCAAATCTGAGATAAGTGCATCTACTTTGTAGAC-3' and 5'-GTCTACAAAGTAGATGCACTTATCTCAGATTTGAGTTAATTATCGGATTTATCTTCTGGCGTCTTACCCATCC-3'.
pCS2-*Xenopus*dnRobo3-MT and pCS2-Igk-ratdnRobo2-MT (Hocking et al., 2010) were kind gifts from Sara McFarlane (University of Calgary, Canada). pCS2-ratdnRobo2-MT used in this study was obtained from pCS2-Igk-RatdnRobo2-MT by removal of Igk and rat Robo2 5’UTR sequences.

Quality, quantity and purity of axonal RNA. The quality and quantity of the RNA was determined by Bioanalyzer (Agilent 2100) and Qubit (Life Technologies now Thermo Fisher Scientific), respectively. The purity of the axonal RNA was assessed by the presence of β-actin but not MAP2 mRNA or histone H4 mRNA by RT-PCR. DNA oligos used for PCR were the following: β-actin mRNA, 5’- CGTAAGGACCTCTATGCCAA-3’ and 5’- TGCATTGATGACCATACAGTG-3’; MAP2 mRNA, 5’- TCAATGGAGAAATGCCATCA-3’ and 5’- TGAATGGAGGAAGGTCTTGG-3’; Histone H4 mRNA, 5’-GGCAAAGGAGGAAAAGGACT-3’ and 5’-GAGAGCGTACACCACATCCA. 35-40 cycles were performed at 60°C.

small RNA sequencing data analysis. The sequencing results from the sRNA axonal libraries were 3’-adapter trimmed and de-duplicated using the *reaper* and *tally* command-line tools from Kraken (Davis et al., 2013). The processed sRNA-Seq reads were mapped to all available *Xenopus tropicalis, Xenopus laevis* and *Danio rerio* pre-miRNA sequences (miRBase v21, (Kozomara and Griffiths-Jones, 2014)). Read mapping was performed using Bowtie (Langmead et al., 2009). The mapping process was performed in four steps: 1) mapping all reads, allowing 0 mismatches, 2) mapping only reads failing to map in the previous step and longer than 10 nucleotides, allowing 1 mismatch, 3) mapping only reads failing to map in the previous step and longer than 20 nucleotides, allowing 2 mismatches and 4) mapping only longer reads failing to map in the previous step, allowing 3 mismatches. To eliminate redundancy between pre-miRNAs mapped to the same genome location, pre-miRNAs were mapped to the *Xenopus laevis* genome (downloaded from ftp://ftp.xenbase.org/pub/Genomics/JGI/Xenla6.0/). Pre-miRNAs mapped to *Xenopus laevis* with identity < 80% were discarded. Many pre-miRNAs could map to the same genome location with identity > 80%, so we ordered them according to identity and kept the pre-miRNA with the highest

score. We did not impose a species name preference for identity ties, we simply kept the first one. Quantification of mapped reads that overlapped with non-redundant pre-miRNAs and mature miRNA regions was performed using the R package *GenomicRanges* [R Core Team (2015). R: A language and environment for statistical computing. R Foundation for Statistical Computing, Vienna, Austria. URL http://www.R-project.org (Lawrence et al., 2013)]. The minimum overlap required between mapped reads and mature regions was 16 nt. We collapsed mature counts by mature miRNA id regardless of species name. All RNA-seq data are deposited in Gene Expression Omnibus (GEO) datasets under accession number GSE86883.

miR-182 Target prediction: Microarrays from *Xenopus laevis* growth cones were downloaded from GEO (GSE25166), and normalized together using *vsnrma* from the vsn R package (Huber et al., 2002). The average expression of the stage 32 growth cone (grown in culture for 24h, thus corresponding to in vivo stage 37/38) samples (GSM618289, GSM618290, GSM618291, GSM618292) was calculated. *Xenopus laevis* gene models (1.8.3.2) and their annotation on the *X. laevis* v9.1 genome were downloaded from Xenbase 4.0 (http://www.xenbase.org/, RRID:SCR_003280) (James-Zorn et al., 2015; Karpinka et al., 2015). The 3'UTR sequences from all protein-coding transcripts were obtained using a custom R script with Biostrings (Pages H. et al. 2016) and bedtools (Quinlan and Hall, 2010). TargetScan Context+ scores were calculated for all miR-182-5p potential targets using the perl script downloaded from http://www.targetscan.org/vert_61/vert_61_data_download/targetscan_61_context_scores.zip (Garcia et al., 2011). Transcripts with the same Gene Symbol (ignoring L/S variants) were collapsed, selecting the one with the best Context+ score. Genes without any assigned GO term, or not represented on the Affymetrix *Xenopus laevis* Genome Array were excluded from further analyses. Finally, the stage 32 growth cone expression of each gene was combined with the TargetScan Context+ score, using the TargetExpress method (Ovando-Vazquez et al., 2016). This method allows to prioritize predicted targets that are likely to be functional based on their expression level in the cell type of interest.

Growth cone turning assay. Gradients of Slit2 recombinant protein (R&D) were established as described in (Campbell and Holt, 2001) by ejecting 10 µg/mL Slit2-containing medium or control medium out of a micropipette with a tip opening of 1µm. Single growth cones, isolated from their cell bodies, from stage 35/36 retinal explants cultured for 24h were positioned 100µm from the tip opening at an angle of 45° relative to the initial direction of the axon shaft. Pictures were taken every 5 min for 1 hr. Turning angles were measured using ImageJ software.

Horseradish peroxidase (HRP) axon tracing. The right lens of anesthetized stage 40 embryos was removed. A paste made of 1% (wt/vol) L-α-lysolecithin (Calbiochem) containing HRP (Roche) was applied in the lensless ocular cavity. After 1h, embryos were fixed in 4% PFA, brains were dissected out and incubated with a 3,3′-diaminobenzidine tetrahydrochloride (Sigma) in 0.1% H_2O_2 solution to reveal HRP staining in RGC axons. Post fixation, ISH for Slit2 was performed, as described previously (Piper et al., 2006). Brains were mounted in lateral view and imaged on a Zeiss Axioskop microscope.

Live imaging of Kaede-cofilin1-3′UTR translation reporter and analysis. Briefly, after initial imaging, Kaede-green is photoconverted to Kaede-red by 6-8 sec exposure to UV light. Axons were then stimulated with suboptimal concentration of Slit2 (125ng/mL) to prevent growth cone collapse, and images were acquired every 5 min on a Nikon Eclipse TE2000-U inverted fluorescence microscope for 30 min. For the analysis, the outline of the growth cone was manually traced in ImageJ software to define a ROI, and both Kaede-green and Kaede-red fluorescence intensity were measured. All measurements were performed on growth cones with non-saturated signal.

Collapse assay. Retinal explants were bathed in 200ng/ml human recombinant Slit2 (R&D) for 10 min with or without cyclohexamide and then fixed in 2% PFA, 7.5% (wt/vol) sucrose. To avoid subjective bias, all collapse analysis was done blind to experimental condition. Growth cones were considered collapsed when they possessed two or fewer filopodia each shorter than 10µm.

Western Blot. The head of anesthetized stage 37-38 embryos were dissected, homogenized and lysed in RIPA buffer (Sigma) containing cOmplete Mini EDTA-free protease inhibitor cocktail (Roche). Centrifugation at 4°C was performed to clear the lysates. Western-Blot was done using rabbit anti-Slit2 (1/500, Genetex) or mouse anti-α-tubulin (1/5000, Sigma), and horseradish peroxidase-conjugated anti-rabbit or anti-mouse secondary antibody (1/5000, Abcam).

RT-PCR for Sensor construct in axons. Control-Sensor electroporated eyes were dissected at stage 37/38 and axons were collected by laser capture microdissection from stage 40 cryosections. Total RNAs were subsequently extracted with Norgen Total (for eyes) or single cell (for axons) RNA Purification Kit and retrotranscribed with SuperScript IV (ThermoFisher) using oligo(dT) primers. The cDNA obtained was amplified for 40 cycles using the following primers: (5'-CACATGAAGCAGCACGACTT-3' Fw d2GFP, 5'-TGCTCAGGTAGTGGTTGTCG-3' Rv d2GFP, and (5'-CCTGTCCCCTCAGTTCATGT-3' Fw mCherry, 5'-CTTCAGCTTCAGCCTCTGCT-3' Rv mCherry.

ISH.
Whole-mount miRNA ISH: miRNA ISH were performed as previously described (Wienholds et al., 2005) on stage 38 or 40 fixed embryos using 2nM DIG-labelled LNA oligonucleotide probes.
miRNA ISH on GCs: Fluorescent ISH (FISH) for miRNA was performed on 24h stage 35/36 retinal cultures with a protocol adapted from (Han et al., 2011). Hybridization was performed with 10-15nM DIG-labelled LNA oligonucleotide probes at 22°C below the predicted Tm. After extensive washes, the ISH signal was detected using anti-digoxygenin antibody conjugated to HRP (Roche) and the fluorescence signal generated by Cy3-tyramide amplification system (PerkinElmer). Retinal cultures were then mounted in ProLong Gold Antifade mountant. FISH signal in RGC GCs was imaged using an Olympus FV1000 confocal microscope equipped with GASP detectors.
miRNA ISH on retinal sections: ISH was carried out on transverse cryosections or vibratome section of stage 40 fixed embryos, as previously described (Baudet et al., 2012; Obernosterer et al., 2007).

Immunostaining. 12μm thick transverse cryosections of fixed stage 40 embryos or stage 35/36 retinal cultures were stained with the following antibodies. Mouse anti–Islet-1 (40.2D6, 1:500, Developmental Studies Hybridoma Bank), rabbit anti-Sox2 (1:500, Millipore) and rabbit anti-cofilin1 (1/200, kindly provided by J. Bamburg). Secondary antibodies were goat anti-mouse Alexa Fluor 488– and goat anti-rabbit Alexa Fluor 555–conjugated F(ab')2 fragments (Invitrogen). Sections were counterstained with DAPI.

Supplemental References

Baudet, M.L., Zivraj, K.H., Abreu-Goodger, C., Muldal, A., Armisen, J., Blenkiron, C., Goldstein, L.D., Miska, E.A., and Holt, C.E. (2012). miR-124 acts through CoREST to control onset of Sema3A sensitivity in navigating retinal growth cones. Nat Neurosci 15, 29-38.

Campbell, D.S., and Holt, C.E. (2001). Chemotropic responses of retinal growth cones mediated by rapid local protein synthesis and degradation. Neuron 32, 1013-1026.

Davis, M.P., van Dongen, S., Abreu-Goodger, C., Bartonicek, N., and Enright, A.J. (2013). Kraken: a set of tools for quality control and analysis of high-throughput sequence data. Methods 63, 41-49.

De Pietri Tonelli, D., Calegari, F., Fei, J.F., Nomura, T., Osumi, N., Heisenberg, C.P., and Huttner, W.B. (2006). Single-cell detection of microRNAs in developing vertebrate embryos after acute administration of a dual-fluorescence reporter/sensor plasmid. Biotechniques 41, 727-732.

Garcia, D.M., Baek, D., Shin, C., Bell, G.W., Grimson, A., and Bartel, D.P. (2011). Weak seed-pairing stability and high target-site abundance decrease the proficiency of lsy-6 and other microRNAs. Nat Struct Mol Biol 18, 1139-1146.

Han, L., Wen, Z., Lynn, R.C., Baudet, M.L., Holt, C.E., Sasaki, Y., Bassell, G.J., and Zheng, J.Q. (2011). Regulation of chemotropic guidance of nerve growth cones by microRNA. Mol Brain 4, 40.

Huber, W., von Heydebreck, A., Sultmann, H., Poustka, A., and Vingron, M. (2002). Variance stabilization applied to microarray data calibration and to the quantification of differential expression. Bioinformatics 18 Suppl 1, S96-104.

James-Zorn, C., Ponferrada, V.G., Burns, K.A., Fortriede, J.D., Lotay, V.S., Liu, Y., Brad Karpinka, J., Karimi, K., Zorn, A.M., and Vize, P.D. (2015). Xenbase: Core features, data acquisition, and data processing. Genesis 53, 486-497.

Karpinka, J.B., Fortriede, J.D., Burns, K.A., James-Zorn, C., Ponferrada, V.G., Lee, J., Karimi, K., Zorn, A.M., and Vize, P.D. (2015). Xenbase, the Xenopus model organism database; new virtualized system, data types and genomes. Nucleic Acids Res 43, D756-763.

Kozomara, A., and Griffiths-Jones, S. (2014). miRBase: annotating high confidence microRNAs using deep sequencing data. Nucleic Acids Res 42, D68-73.

Langmead, B., Trapnell, C., Pop, M., and Salzberg, S.L. (2009). Ultrafast and memory-efficient alignment of short DNA sequences to the human genome. Genome Biol 10, R25.

Lawrence, M., Huber, W., Pages, H., Aboyoun, P., Carlson, M., Gentleman, R., Morgan, M.T., and Carey, V.J. (2013). Software for computing and annotating genomic ranges. PLoS Comput Biol 9, e1003118.

Obernosterer, G., Martinez, J., and Alenius, M. (2007). Locked nucleic acid-based in situ detection of microRNAs in mouse tissue sections. Nat Protoc 2, 1508-1514.

Ovando-Vazquez, C., Lepe-Soltero, D., and Abreu-Goodger, C. (2016). Improving microRNA target prediction with gene expression profiles. BMC Genomics 17, 364.

Pages H. et al., 2016. Biostrings: String objects representing biological sequences, and matching algorithms. R package version 2.40.2. (https://bioconductor.org/packages/release/bioc/html/Biostrings.html).

Piper, M., Anderson, R., Dwivedy, A., Weinl, C., van Horck, F., Leung, K.M., Cogill, E., and Holt, C. (2006). Signaling mechanisms underlying Slit2-induced collapse of Xenopus retinal growth cones. Neuron 49, 215-228.

Quinlan, A.R., and Hall, I.M. (2010). BEDTools: a flexible suite of utilities for comparing genomic features. Bioinformatics 26, 841-842.

Wienholds, E., Kloosterman, W.P., Miska, E., Alvarez-Saavedra, E., Berezikov, E., de Bruijn, E., Horvitz, H.R., Kauppinen, S., and Plasterk, R.H. (2005). MicroRNA expression in zebrafish embryonic development. Science 309, 310-311.
